# Fractionation of Kraft Lignin for Production of Alkyd Resins for Biobased Coatings with Oxidized Lignin Dispersants as a Co-Product

**DOI:** 10.1021/acsomega.4c07187

**Published:** 2024-11-04

**Authors:** Arpa Ghosh, Olesya Fearon, Melissa Agustin, Susana Alonso, Estefanía Cámara Balda, Saulo Franco, Anna Kalliola

**Affiliations:** † VTT Technical Research Centre of Finland Ltd., P.O. Box 1000, FI-02044 Espoo, Finland; ‡ Barpimo S.A., Calle San Fernando, 116, 26300 Nájera, La Rioja, España

## Abstract

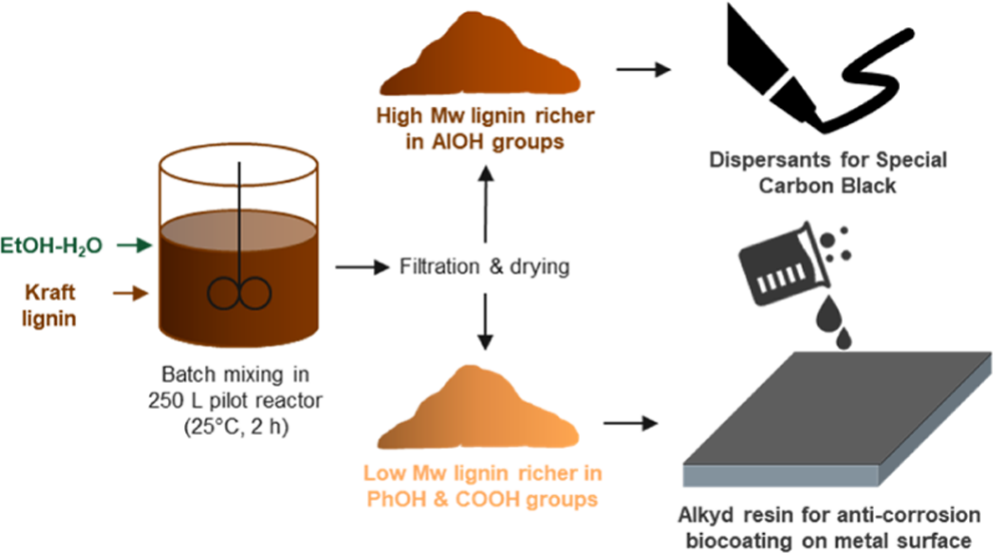

A new valorization pathway based on solvent fractionation was applied to kraft lignin, a major by-stream of the pulping industry, to extract a soluble lignin intermediate featuring an improved structural homogeneity, a low molecular weight, and a high content of phenolic hydroxyl and carboxylic acid groups to serve as a substitute of the nonrenewable polyacids in the formulation of alkyd resins, a dominant material used in the production of anticorrosion surface coatings. Herein, softwood kraft lignin was mixed in a low-cost green solvent, aqueous ethanol, prepared at different ratios, at room temperature to generate a soluble fraction of a low *M*_w_ of ≤2200 g mol^–1^ and an insoluble fraction of a high *M*_w_ of ≥3950 g mol^–1^ of lignin. The best combination of yields and molecular weights of soluble lignin (16–36% yield, 1740–1890 g mol^–1^) was attained using 50–80 vol % ethanol in fractionation. Thus, these conditions were further employed at the pilot scale to demonstrate the scalability of this technology. Soluble lignin from pilot fractionation was used to produce an optimal alkyd resin formulation and thereafter an anticorrosion coating on the metal surface, both of which matched the target properties of industrial standards well (180 s Persoz hardness and 72 gloss units of coating, 100% adhesion of paint with no cracks or peeling in the cross-cut test, no corrosion after 120 h of the salt spray test). The insoluble solids from pilot fractionation could also be valorized by alkali-O_2_ oxidation into lignin-based dispersants for special carbon black pigments. Overall, this study presents a new, simple strategy to develop an efficient, scalable, low-cost, and green process for upgrading kraft lignin into phenolic intermediates for biobased alkyd resins to facilitate sustainable production of high-performance anticorrosion coatings.

## 1 Introduction

Lignin is a highly abundant renewable source of natural biopolymers that can serve as a promising resource of sustainable materials to replace everyday fossil-based products. Although various biorefining processes such as kraft pulping, soda pulping, organosolv extraction, and enzymatic hydrolysis can fractionate lignin from the lignocellulosic feedstock, kraft pulping is still the predominant source of industrial lignin.^1,2^ At present, in kraft pulp mills, black liquor with kraft lignin is mostly burnt to generate bioenergy and recover pulping chemicals.^3^ However, recently, advanced technologies such as LignoBoost have managed to extract kraft lignin from black liquor at a quantity surpassing the energy demand of the pulp mills and generating up to 100,000 tons of commercially available lignin globally per year.^4,5^ A variety of valorization opportunities for kraft lignin into high-value bioproducts has been explored in the recent years including some applications at the industrial scale.^6^ For example, the use of kraft lignin as a phenol replacement in phenol formaldehyde resins has been commercialized for plywood and laminates while sulfonated lignin has been used as dispersants commercially, similar to lignosulfonates.^7,8^ Nevertheless, despite the emergence of these important petroleum-alternative solutions, the market of large-volume products from kraft lignin is still under development.^6^ Undoubtedly, since kraft lignin production has already been commercialized in recent times, this significant technological development would be more beneficial from the standpoint of economics and environmental impact if more of the value-added bioproducts derived from kraft lignin can be produced at an industry-relevant scale.

Kraft lignin is a polyphenolic material containing plenty of both phenolic and aliphatic hydroxyl groups along with carboxyl acid functionalities.^9^ Accordingly, this underutilized resource has been identified as a renewable, sustainable alternative to petroleum-derived phenol, polyol, and polyacid intermediates that are the main platform chemicals used in the formulation of resins for application in paints and coatings.^10^ Alkyd resins as binders dominate the paint and coating industry owing to their superior performance (good aging, greater weather resistance, high heat resistance, outstanding gloss, etc.), easy application, low cost, and versatility of use.^11^ Currently, approximately 200,000 tons of alkyd resins are produced each year^12^ and the market of these resins is estimated to grow up to 5.3 billion USD by 2030.^13^ The alkyd resin has a polyester backbone, which is synthesized by the reaction of polyols, such as glycerol, and polyacids (acid anhydrides, diacids, and fatty acids), such as phthalic anhydride.^11^ While some polyols and fatty acids used in the synthesis of alkyd resins can come from renewable sources like vegetable oils, the acid anhydrides or diacids are still dependent on petroleum-based chemicals.^14^ In addition, these raw materials can have a high cost, for example, 1800 euros for a ton of phthalic anhydride, and the properties of alkyd resins made from renewable oils may not be satisfactory.^15^ Therefore, substituting petroleum-derived polyacids and/or polyols in the formulation of alkyd resins effectively by kraft lignin will be a great step toward decarbonizing the coating industry. Furthermore, the polyphenolic structure of kraft lignin could also help improve the mechanical strength and resistance to ultraviolet (UV), water, fungal attack, and lower volatile organic compound emissions, leading to a superior quality of the alkyd surface coating.^16^

Pathways for valorization of kraft lignin have been studied extensively with a major focus on polyurethanes, epoxies, and phenol formaldehyde resins.^17^ However, reports on lignin-based alkyd resin are scarce in the literature. Despite having many phenolic hydroxyl and carboxylic acid groups, using kraft lignin as a raw material replacement in any resin formulation faces several challenges. The structure of kraft lignin is highly diverse, featuring a very broad molecular weight distribution, high dispersity, and presence of a variety of functional groups, branching, and chemical bonds.^9^ To utilize this lignin material efficiently in the production of resins from phenolic polyacids and/or polyol intermediates, lignin needs to be turned into a chemical intermediate having a uniform distribution of low molecular weight.^18,19^ Catalytic depolymerization and chemical functionalization of kraft lignin could be employed to break down the complex polymeric structure of lignin into homogeneous phenolic monomers and oligomers or modify by incorporating chemical groups for an enhanced quality of resin and coating materials.^10,17^ However, these processes often use high temperature and pressure, hazardous chemicals, and solid catalysts requiring cost-intensive reactor and purification units and thus could often be less attractive routes at the industrial scale.

Solvent fractionation of kraft lignin is a great alternative pathway to reduce the heterogeneity of lignin by producing different fractions of lignin with low and high molecular weights.^20^Table 1 summarizes the state-of-the-art solvent fractionation processes for valorization of lignin including coating applications. This process typically uses an organic solvent, as pure or mixed with another solvent or water, to solubilize lignin mostly at room temperature in different components according to different molecular weights.^21^ This helps in producing a kraft lignin fraction of a lower molecular weight and improved uniformity in physical and chemical properties than the parent lignin. In addition, the mild process conditions, simplicity of the process, and requirement of minimal cost-effective separation steps are some of the key advantages of this fractionation method.

**Table 1:** Literature Review on Solvent Fractionation of Kraft Lignin with Application in Coatings

lignin	solvent	fractionation method	fractionation conditions	scale	lignin product yield	solvent effects on process efficiency/economics/sustainability	solvent effects on the quality of the lignin product	structure–property performance relationship	application	country, year^ref^
hardwood kraft lignin	acetone, Et_2_O, EtOH, hexane	one-step solid–liquid extraction in each solvent	mixing at RT for 2 h	lab	70–80% soluble lignin	N/A	highest *M*_w_ in EtOH and lowest *M*_w_ in Et_2_O, high solubility in highly polar solvents	N/A	N/A	Slovenia, 2021^30^
softwood kraft lignin (LignoBoost)	acetone, EtOH, EtOAc, IPA, MeOH, MEK	one-step solid–liquid extraction in each solvent	mixing at different solid/liquid ratios	lab	50% soluble lignin (max)	soluble fraction yield increased with an increasing liquid/solid ratio	N/A	linear correlation of Tg vs Mn, soluble lignin high in phenolic OH content	N/A	USA, 2020^27^
softwood kraft lignin (LignoBoost)	acetone, EtOAc, MeOH as the pure/aqueous mixture/solvent mixture	two-step solid–liquid extraction in the solvent	mixing at RT for 2 h	lab	7.5–66% soluble lignin (pure solvent), 44.1–98.5% soluble lignin (mixtures)	soluble fraction yield decreases as the number of carbon atoms increases for alcohols and ketones, high ratio of solvent/water increases the soluble lignin yield, the acetone–water mixture was most energy-efficient, cost-effective, and environmentally friendly (50 ton/d scale model)	solvent mixtures are robust for controlling the molecular weights and reducing dispersity by up to 73%	N/A	N/A	Sweden, Canada, 2019^31^
softwood kraft lignin (Indulin AT)	2-butanone, MeOH, THF	one-step Soxhlet extraction in each solvent	mixing at 100 °C (2-butanone), 85 °C (MeOH), 86 °C (THF) for 8 h	Lab	21–62% soluble lignin	soluble fraction yield in THF > MeOH > 2-butanone, the Flory–Huggins polymer–solvent interaction parameter correlated with yields, H-bonding between lignin and the solvent plays a key role	*M_n_* and PDI of soluble lignin lowest in 2-butanone and highest in MeOH, relative content of hydroxyl and carbonyl groups in the soluble fractions well correlated with the H-bonding ability of the solvent (highest for MeOH)	linear correlation of the Tg and the char mass residue (TGA) with the Mw of soluble lignin	N/A	Italy, 2016^32^
hardwood kraft lignin	acetone, dioxane/water 95:5%, EtOAc, MEK, MeOH	multistep sequential extraction in 5 solvents	mixing at RT for 2 h	lab	2.9–51.1% soluble lignin (90.7% total soluble lignin)	extraction yields of soluble lignin fractions vary due to different solubilities in organic solvents	*M*_w_ distribution increased from the fraction 1 to 5	higher heating value is inversely proportional to the *M*_w_, low *M*_w_ fractions show a low carbonaceous residue	N/A	Brazil, 2020^33^
enzymatic hydrolysis lignin	*n*-ButOH, DCM, EtOAc	multistep sequential extraction in 3 solvents	ultrasound-assisted mixing at RT for 2 h	lab	13.4–46.2% soluble lignin (F1–F4)	yield of soluble lignin lowest in F1, highest in F4	lowest *M*_w_ and highest phenolics in F1	highest antioxidant activity in F1	N/A	China, 2017^34^
wheat straw soda lignin (SSL), organosolv lignin (OSL), softwood kraft lignin (SKL)	acetone/water	multistep sequential precipitation in acetone/water (60 and 40%)	mixing at RT for 1 h	lab	34.8% soluble lignin	yield of soluble lignin highest for SKL ∼ SSL > OSL	fractions with low *M*_w_ dissolved in solutions with a greater proportion of water, higher amount of phenolic hydroxyl groups and carboxylic acid groups in lower *M*_w_ fractions, fractions of high purity and homogeneity but the structure and composition depend on the source of lignin	N/A	N/A	Finland, 2018^35^
organosolv lignin	acetone/water	multistep sequential precipitation (60 to 30%)	mixing at RT for 1 h	lab	>75% soluble lignin	maximum yields of soluble lignin in 45 and 55% acetone	decreasing *M*_w_ and high phenolic OH with decreasing acetone % in the solvent, higher molecular weight fractions showed more phenolic and less aliphatic OH	fractions of lower *M*_w_ with a higher amount of phenolic OH show higher antioxidant activity	N/A	USA, 2017^36^
softwood kraft lignin	acetone, EtOH, PGME	multistep sequential precipitation (reducing solvent concentration in water)	mixing at RT for 20 min in each of 10 steps	lab	84% (60% acetone), 68% (80% EtOH), 99.2% (60% PGME) soluble lignin	high solubility in PGME and acetone, but higher stability of soluble lignin in EtOH against precipitation by water, gradient mixing of the solvent enables an easy tuning of the process and selection of precipitation steps depending on the purity, molar mass, and solubility requirements	decreasing *M*_w_ with the decreasing solvent concentration, high *M*_w_ fractions have low free phenolic OH and COOH, low *M*_w_ fractions have high phenolic OH	N/A	N/A	Finland, 2017^26^
softwood kraft lignin (Indulin AT), soda lignin (Protobind)	alkaline EtOH/water (60:40%), MEK	solubilization in aqueous EtOH or MEK followed by sequential membrane ultrafiltration	Soxhlet extraction/cascade membrane ultrafiltration	lab	75–80% soluble lignin	high solubility in MEK and 60:40% alkaline EtOH/water	low *M*_w_ fractions have high phenolic OH, ultrafiltration enabled isolation of fractions with narrowly controlled chemical, structural, molecular, and thermal characteristics irrespective of the biomass origin and solvents used	N/A	N/A	Italy, 2020^37^
softwood kraft lignin (Indulin AT)	MeTHF	one-step Soxhlet extraction	mixing at 80 °C for 8 h	lab	50% MeTHF-soluble lignin	uses a bioderived solvent system	lower *M*_w_ lignin fraction with lower dispersity, slightly lower concentration of OH groups	high Tg values of the PU materials prepared from MeTHF-soluble lignin, cross-linked PU films with moderate hydrophobicity, coarser morphology in PU films at a higher lignin content, PU films have a 2–4 GPa elastic modulus and a good adhesive performance on wood	PU thermoset films	Italy, 2015^38^
softwood kraft lignin	acetone	one-step solid–liquid extraction in the solvent	mixing at RT for 12 h	lab	70% acetone-soluble/30% acetone-insoluble lignin	uses a non-VOC solvent (acetone), acetone-soluble lignin used to make the phenolic resin replacing 100% of phenol, acetone-insoluble lignin used to make the PU resin replacing 100% of petroleum-based polyol	acetone-soluble lignin is rich in phenolic OH content, acetone-insoluble lignin is rich in aliphatic OH and show higher reactivity toward isocyanate, fractionation in acetone improved homogeneity, ash content high in insoluble lignin	higher dry adhesion strength than the phenol formaldehyde resin, PU resin made in the Cyrene solvent shows hardness and flexibility (lower Tg) and lower viscosity	wood coatings and adhesives	USA, 2022^19^
biorefinery lignin (byproduct of bioethanol production from corncobs)	Bio-EtOH	one-step solid–liquid extraction in the solvent	mixing at RT for 1 h	lab	50% EtOH-soluble lignin	uses a biorenewable green solvent (ethanol)	fractionation reduced the *M*_w_ and dispersity, fractionation and oxypropylation improved the reactivity of lignin with isocyanates	the fractionated lignin-based PU coating showed a homogeneous coat with a smooth surface, lighter color, and enhanced anticorrosion property on aluminum.	PU coating for aluminum	USA, 2022^28^
softwood kraft lignin (Indulin AT)	THF	one-step Soxhlet extraction in the solvent	mixing at 86 °C for 8 h	Lab	65% THF-soluble lignin	uses a fossil-based toxic, hazardous solvent (THF)	lower *M*_w_ and dispersity after fractionation	silanized THF-soluble lignin had better thermal stability and hydrophobicity than the nonsilanized one, adhesion to aluminum improved with addition of a cross-linker promoter (TEOS) in the formulation.	PU coating for aluminum	Italy, 2019^39^
softwood kraft lignin	acetone–MeOH (70:30%) cosolvent/hexane as the antisolvent	multistep sequential precipitation	mixing at RT for 30 min	lab	8.6–19.8% precipitated lignin fractions	yield of soluble lignin could be tuned by precipitation with increasing hexane vol %	produced high (54k), medium (15k), and low (4k) *M*_w_ fractions, *M*_w_ and dispersity lowered with increasing hexane vol %, secondary polyol blending needed to enhance reactivity and solubility in THF	increasing the *M*_w_ of the lignin fraction improved the material stiffness or resistance to deformation of PU, blending with secondary polyol (PEG) reduced the brittleness and enhanced the ductility of PU	PU films for the coating	USA, 2019^40^
technical lignin	acetone, EtOAc, MEK, MeOH, and mixtures of any of these with water	semicontinuous solid–liquid extraction	solvents flush through successively in the packed bed column with lignin and inert particles	patented (large scale)	10–27% lignin of *M*_w_ < 3000 g/mol	increased speed of extraction of the most desired lower *M*_w_ fractions from technical lignins, time and cost-effective process	lower *M*_w_ and dispersity, higher content of functional groups and reactivity, lower viscosity of lower *M*_w_ fraction	higher quality for applications as wood adhesives, polyol substitution in PU foams and coatings	wood adhesives, PU foams	worldwide, 2015^41^
kraft lignin	acetone, acetonitrile, DMF, DMSO, alcohols, etc. with water	solid–liquid extraction with successive ultrafiltration	dissolution at 50–120 °C	patented	30–90% soluble lignin	uses toxic, hazardous solvents with complicated recovery methods	different *M*_w_ fractions obtained by ultrafiltration steps (50, 15, and 5 kDa) for different applications	N/A	adhesives for wood veneer, additives for thermosets and thermoplastics	US/Finland, 2018^42^
organosolv lignin	60 to 15% acetone/water as the antisolvent	multistep sequential precipitation followed by reductive partial depolymerization	precipitation at RT for 30 min	pilot (0.5 kg in 5 L)	21–27% precipitated lignin fractions	yield of different lignin fractions could be tuned by precipitation with increasing water vol %	lower *M*_w_ and dispersity, higher phenolic OH content, and lower abundance of lignin interunit linkages with decreasing acetone vol %	high-*M*_w_ lignins gave coatings that had a high Tg and were more rigid and hydrophobic, low-*M*_w_ lignins led to PU coatings with a lower Tg, more hydrophilic behavior, and enhanced flexibility	PU coatings	Italy, Netherlands, 2023^43^

Most studies on solvent fractionation have been performed on a laboratory scale with the aim of determining the relationship of solvent properties with the yields and characteristics of lignin fractions as well as any structure–property performance relationship for lignin-based bioproducts (see Table 1). Generally, it is observed that high-polarity solvents with a strong H-bonding capability help in greater solubilization of kraft lignin.^22^ Solvent fractionation of lignin can be achieved by one-step solid–liquid extraction in one or more organic solvents or a multistep sequential precipitation by an antisolvent, mainly water. A soluble lignin fraction of low molecular weight (below 3000 g mol^–1^) can be produced at 50–80% yields, which can subsequently be used to prepare polyurethane films, wood coatings, adhesives, and anticorrosion films. The research studies listed in Table 1 outline the importance of controlling the molecular weights, dispersity, and concentrations of various chemical functionalities, mainly phenolic hydroxyl groups (phenolic OH), aliphatic hydroxyl groups (aliphatic OH), and carboxylic groups (COOH), in the lignin fractions to attain the desired physicochemical properties of the target resins and coatings.

Despite a plethora of research efforts initiated in the field of solvent fractionation, including some with coating applications, this process is still yet to be commercialized for biobased coating production from kraft lignin. The use of green, safe, biobased, easily recyclable, and low-cost solvents is generally preferred for sustainable development of any biorefining technology.^23^ Ethanol, although a flammable chemical, is often a “preferred/recommended” solvent in chemical processes due to its relatively lower environmental impact.^23^ Additionally, ethanol is cheaper and biobased,^24^ and can be readily separated from aqueous mixtures by distillation (up to 85 wt % purity).^25^ However, as evidenced from Table 1, currently available ethanol-based one-step fractionation methods are not fully developed beyond the laboratory scale.^26,27^ Despite producing lignin fractions with favorable properties for coatings at the laboratory scale, none of these technologies have been applied at large scale for the synthesis of alkyd resins.^28^ On the other hand, multistep ethanol-based fractionation can be difficult to scale up due to inefficient use of water during sequential precipitation of lignin and subsequent use of multiple separation and drying steps for extracting several lignin fractions.^26^ Additionally, many of the studies do not attempt to fully valorize the entire kraft lignin into different valuable products, rendering the process uneconomical.

The aim of this study was to demonstrate an economic and sustainable valorization pathway of kraft lignin by developing a simple, carbon-efficient, and scalable ethanol-based fractionation process for production of biobased alkyd coatings. In this work, we developed a one-step fractionation process wherein kraft lignin was treated in aqueous ethanol under mild conditions to generate two distinct fractions of soluble and insoluble lignin. The effect of the ratio of ethanol and water on yields, molecular weights, and chemical functionalities of kraft lignin fractions was studied to produce high-quality lignin-based intermediates required for alkyd resins. Soluble lignin was hypothesized to have a low molecular weight, low dispersity, and high amount of phenolic OH and COOH groups and thus be suitable for alkyd resin formulation. Furthermore, the separation and drying processes in this study were also reasonably simple, one-step, and cost-effective to ensure that the overall technology is easy to scale up. The above process was tested at the pilot scale followed by valorization of the resulting soluble lignin fraction into coatings synthesized from alkyd resins. To evaluate the full valorization potential of kraft lignin, the insoluble lignin fraction obtained at the pilot scale was further oxidized using our previously developed alkali-O_2_ process^29^ and tested as a dispersion material for carbon black.

## 2 Methods

### 2.1 Materials

#### 2.1.1 Kraft Lignin

The unfractionated lignin material used in this work was dry industrial softwood kraft lignin. Kraft lignin was precipitated from softwood black liquor and was kindly provided by Stora Enso (Lineo). A compositional analysis of kraft lignin was performed as per details given below in Section 2.3.1.

#### 2.1.2 Solvents and Reagents

Ethanol (purity min 94 wt %) was purchased from Anora Group Oyj and was used as the primary solvent in this work. Reverse osmosis water, supplied onsite, was used to prepare aqueous ethanol mixtures for solvent fractionation. The reagents, sodium hydroxide (NaOH) and 37 wt % hydrochloric acid (HCl), were obtained from Merck and Thermo Scientific, respectively.

#### 2.1.3 Raw Materials for Synthesis of the Alkyd Resin and Coating

Trimethylolpropane (purity min 99.5 wt %) was supplied by Biltrec, neopentyl glycol (purity min 99.2 wt %) was supplied by LG Chem, isophthalic acid (purity 99.9 wt %) was supplied by Quimidroga, trimellitic anhydride (purity min 99.5 wt %) was supplied by Biltrec, and dehydrated castor oil fatty acid was supplied by Castor Girnar Industries.

The low-formaldehyde melamine resin was procured from BASF. Xylene, potassium hydroxide (KOH, 0.5 N), tetrahydrofuran (THF), polystyrene standards, phthalic anhydride, drier 2% blend (dry time test), saline solution (5% salt content), the commercial alkyd resin standard, and the commercial oil-based paint standard for the anticorrosion coating were obtained onsite at Barpimo.

### 2.2 Experimental Procedures

#### 2.2.1 Production of Biobased Coatings from Kraft Lignin by Solvent Fractionation

**Figure 1:**
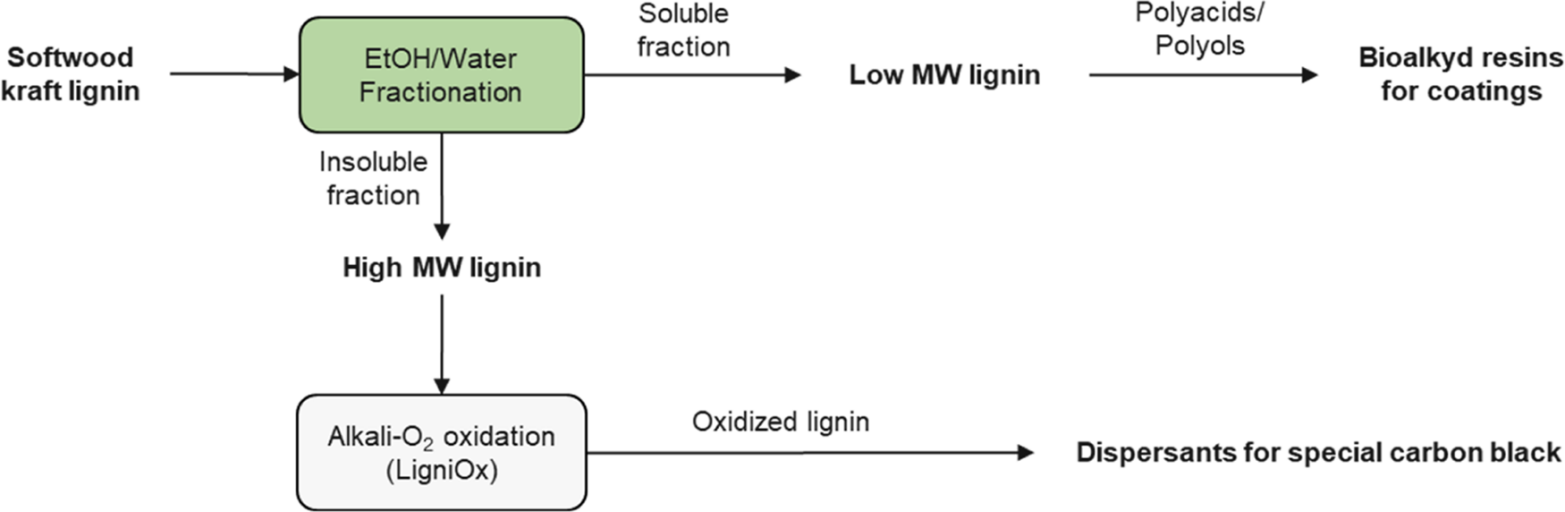
Schematic of the valorization pathway of kraft lignin to bioalkyd resins for coatings via the solvent fractionation process and optional/additional production of the lignin-based dispersant as a byproduct.

Solvent fractionation in a mixture of ethanol/water (EtOH/water) was performed to transform kraft lignin into the intermediate lignin material for the preparation of biobased coatings. More specifically, the fraction of kraft lignin solubilized in aqueous ethanol during the fractionation had a lower molecular weight and was applied in coatings. To fully utilize the starting kraft lignin material, the insoluble fraction of kraft lignin having a higher molecular weight could be converted to a dispersant by applying alkali-O_2_ oxidation, followed by membrane concentration.^44,45^ Notably, the soluble lignin fraction did not need to be oxidized prior to alkyd resin production. Figure 1 shows the overall schematic of the production of bioalkyd resins for coatings from kraft lignin using ethanol/water fractionation and having oxidized lignin as a dispersant byproduct.

The details of each processing step in the above scheme are discussed in the following sections below.

#### 2.2.2 Solvent Fractionation of Kraft Lignin at the Laboratory Scale

Solvent fractionation of kraft lignin was performed at the laboratory scale first to determine the optimum ethanol/water ratio for the process. The above optimum conditions for solvent fractionation were then applied for the pilot-scale fractionation of lignin to produce sufficient material for biobased coating formulations. Lignin was added in the amount of 316 g in a total of 3 L of aqueous ethanol solutions prepared at different ethanol-to-water ratios. The resulting lignin slurry was mixed thoroughly under magnetic stirring at 25 °C (or room temperature) for 2 h. As a next step, the suspension was filtered using a Tamfelt S1114-L2K2 (Metso Fabrics Oy) filter fabric. The insoluble fraction remained as a solid residue on the fabric, while the soluble fraction passed through the fabric and was collected for further analysis. The insoluble fraction was dried in a heating cabinet at 40 °C and weighed. The soluble fraction was concentrated by evaporating the solvent and subsequently freeze-dried prior to weighing. The reproducibility of yields of soluble and insoluble lignin fractions was determined by replicating a selected fractionation condition (50 vol % ethanol), and the results are given in the Supporting Information (Table S1). Standard deviations of yields were within 1 wt % of the mean yields.

#### 2.2.3 Solvent Fractionation of Kraft Lignin at the Pilot Scale

**Figure 2:**
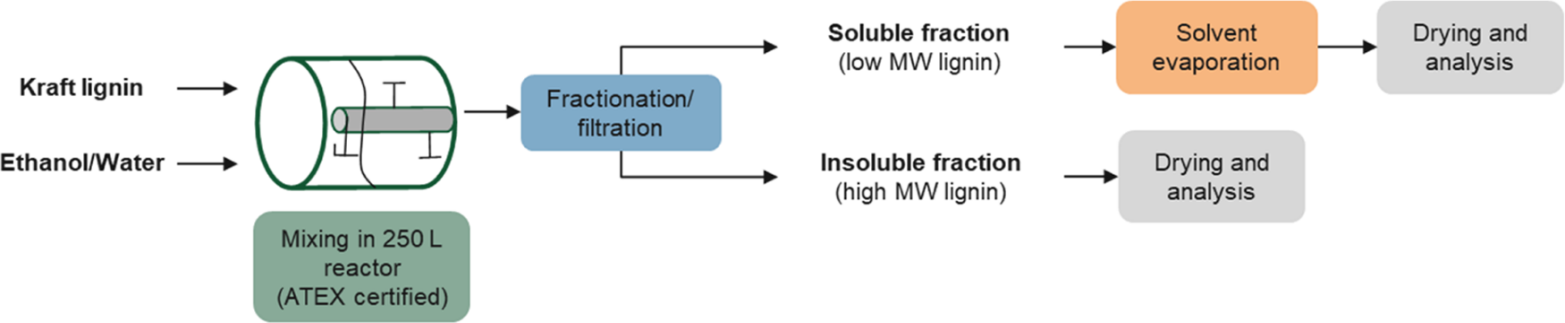
Schematic of the solvent fractionation of kraft lignin on a pilot scale.

The pilot-scale solvent fractionation of kraft lignin was performed at the VTT Bioruukki Pilot Center. Based on the best results from the laboratory scale, lignin fractionation was conducted at three ratios of ethanol and water: 50:50, 65:35, and 80:20 vol % ethanol/water. For safe operations, the pilot-scale solvent fractionation was carried out in ATEX-certified equipment. The reactor setup used for the solvent fractionation of lignin was Drais TurbuDry TD 250 E. As shown in Figure 2, lignin was added (17 kg dry weight) in 160 L of aqueous ethanol and then the slurry was mixed at 25 °C (or room temperature) for 2 h in a 250 L reactor. The suspension of lignin in the solvent was filtered at the end of the mixing process using a Tamfelt S1114-L2K2 (Metso Fabrics Oy) filter fabric. The insoluble fraction was rejected by the fabric, whereas the soluble fraction went through the filter. The insoluble fraction was washed twice with an aqueous ethanol mixture (50:50 vol %), after which it was dried and weighed. The soluble fraction was concentrated by evaporating the solvent and then freeze-dried and weighed. The samples of the pilot-scale fractionation of lignin were distributed to Barpimo for resin preparation to produce alkyd coatings.

Yields of soluble and insoluble fractions of lignin produced at laboratory and pilot scales were termed *Y*_sol_ and *Y*_Insol_, respectively. These yields were determined by eqs 1 and 2 as shown below
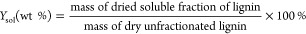
1
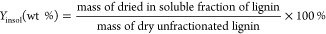
2

#### 2.2.4 Production of the Lignin-Based Alkyd Resin and Anticorrosion Coating on a Metal Surface

**Figure 3:**
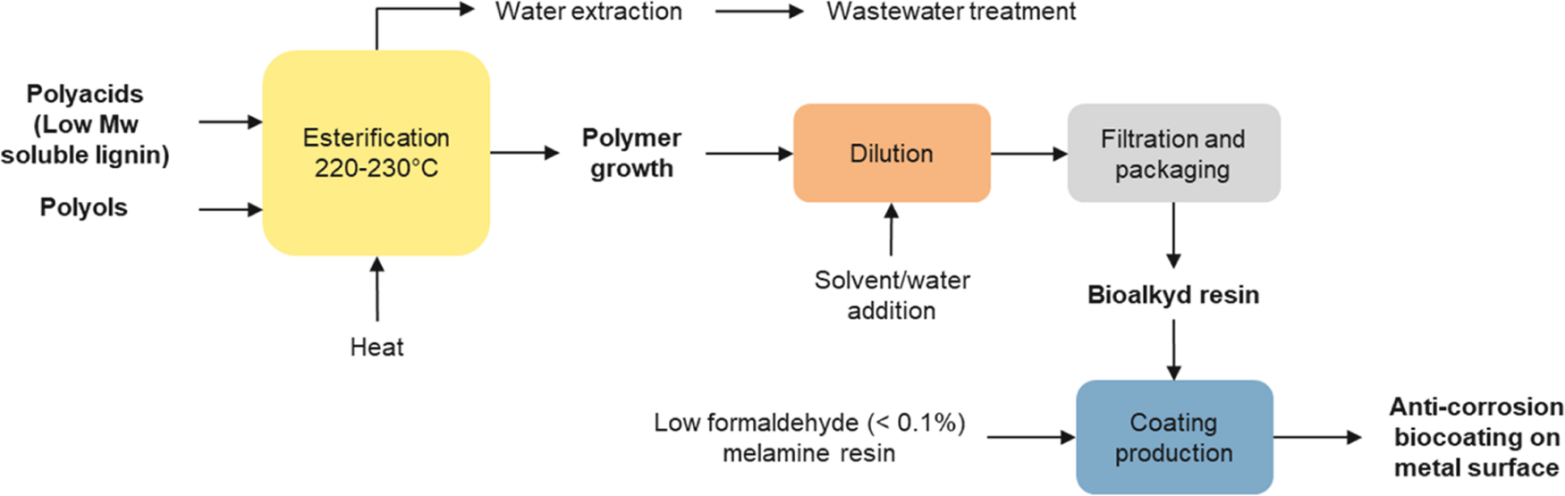
Schematic of the process for production of the lignin-based alkyd resin and anticorrosion coating on the metal surface.

As shown in the Figure 3 schematic, an alkyd resin was prepared in a glass flask reactor equipped with a thermometer, heating mantle, stirrer, and rectification column. A polyacid substrate (either phthalic anhydride or phthalic anhydride partially replaced by the soluble lignin fraction produced by VTT as per the method given in Section 2.2.3), was combined with trimethylolpropane, neopentyl glycol, dehydrated castor oil fatty acid, and isophthalic acid and then charged in the reactor vessel and heated to 220–230 °C in 4 h under reflux. A small amount of xylene was used for the azeotropic distillation (about 1% of the total volume). When the reaction mass reached the temperature, the mixture was held until all water from the reaction was distilled off, and the acid number was less than 10 mg KOH g^–1^. After reaching the indicated acid number, the resin was cooled to 170 °C and then trimellitic anhydride was added to the reactor. The reaction continued until a Gardner viscosity of X-Y (ASTM D1725–12) and an acid number of <35 mg KOH g^–1^ (ASTM D1639–90) were attained. After reaching these parameters, the resin was cooled and dispersed in demineralized water at 42% on solids and then filtered. In the above synthesis, 25% of phthalic anhydride could be substituted by a soluble lignin fraction. The lignin content was 15% in the alkyd resin formulation.

The resin, namely, V004/2023–80% EtOH, was based on the 80% EtOH-soluble lignin fraction, as produced at the pilot scale in VTT, and it was further used to formulate an alkyd biocoating for anticorrosion protection of metals (Figure 3). An oven-dried, water-based, black paint, based on the cross-linking of the low-formaldehyde (<0.1%) melamine resin and the bioalkyd resin V004/2023–80% EtOH, was developed. The properties of the above lignin-based paint were tested and compared with a commercial oil-based paint.

#### 2.2.5 Production of Dispersants for Special Carbon Black from the Insoluble Lignin Fraction

Alkali-O_2_ oxidation was applied to convert the insoluble lignin fraction into an oxidized lignin material of high molecular weight, following the previously developed method at VTT by Kalliola et al. in 2022.^45^ In short, the insoluble fraction was dissolved in NaOH at 6% lignin content and oxidized in a pressure vessel under an O_2_ overpressure to produce a dispersant product for special carbon black (CB). The test procedure used in this work was according to a method developed by Fearon et al. at VTT in 2021.^44^ Shortly, CB dispersions were prepared from special carbon black (Printex 60 by Orion Engineered Carbons) with a concentration of 15 wt %. The dispersions were prepared by efficiently mixing the required amount of water, CB, and a dispersant. Dispersant dosages of 5–30 wt % (based on active matter of the dispersant) were used. The performance of oxidized lignin and the reference dispersant (a commercial lignosulfonate-based product) in a CB suspension was evaluated based on the viscosity, ζ-potential, and particle size measurements. It is important to note that oxidation was not optimized for the insoluble fractions. Additionally, no membrane filtration post-treatment was performed on the oxidized lignin solutions.

### 2.3 Analytical Methods

#### 2.3.1 Compositional Analysis of Kraft Lignin Fractions

Compositional analysis of kraft lignin, the unfractionated material used in this work, was performed at VTT to determine the content of dry matter, ash, extractives, lignin, carbohydrates, and protein in the starting material. Dry matter of the lignin material was determined gravimetrically at 105 °C as per the SCAN-N 22:96 method. The ash content was determined gravimetrically after combustion of the samples at 550 °C. The lignin content was a sum of insoluble Klason lignin and acid-soluble lignin content. Klason lignin content was determined gravimetrically after acid hydrolysis (NREL procedure).^46,47^ The hydrolysate from this process was further analyzed to determine the content of acid-soluble lignin using UV spectroscopy^48^ and carbohydrates using HPAEC-PAD.^49^ Elemental analysis (C, H, N, S, O) of the dried (105 °C overnight) unfractionated lignin material was performed using a FLASH 2000 series elemental analyzer (Thermo Scientific, Bremen, Germany). The protein content in unfractionated lignin was computed by multiplying the nitrogen content by 6.25 as the factor. The results of the above compositional analysis of unfractionated kraft lignin are presented in Section 3, Table 2 with additional analytical data provided in the Supporting Information (Tables S2–S3). Dry matter and ash contents of soluble and insoluble lignin fractions obtained after ethanol/water fractionation were analyzed by the same methods as described for unfractionated lignin. The compositions of lignin, carbohydrate, protein, and extractives were not analyzed for these materials.

#### 2.3.2 Determination of the Molecular Weight Distribution of Kraft Lignin Fractions

The molecular weights of the unfractionated kraft lignin material and its soluble and insoluble fractions obtained from solvent fractionation were determined by size exclusion chromatography (SEC) following a method described elsewhere.^26^ The measurements of the molecular weight and dispersity of samples were replicated twice for selected samples to determine the standard deviations of these measurements. The numerical values of these measurements are given in the Supporting Information (Table S4), with chromatographs of the molecular weight distribution of fractionated lignin samples, as shown in Figure S1.

#### 2.3.3 Determination of Chemical Functionalities of Kraft Lignin Fractions

The functional groups were determined by the ^31^P NMR method (nuclear magnetic resonance) developed by Granata and Argyropoulos earlier.^50^ The NMR instrument used in this work was a Bruker 500 MHz NMR spectrometer with the following parameters: 1024 scans, 5 s pulse delay, 90° pulse and line broadening of 2, and default baseline correction. The numerical measurements with standard deviations are given in the Supporting Information, as Table S5 for laboratory-scale fractionation and Table S6 for pilot-scale fractionation. The ^31^P NMR spectra of unfractionated kraft lignin are given in the Supporting Information (Figure S2).

#### 2.3.4 Characterization of Lignin-Based Alkyd Resins and Anticorrosion Coatings

##### 2.3.4.1 Determination of the Molecular Weight Distribution of Alkyd Resins Modified with Kraft Lignin

The molecular weights of the alkyd resin were determined by the gel permeation chromatography (GPC) method. In this method, the GPC Model AZURA Knauer system served as the SEC equipment, which contained a MesoPore 3 μm (300 mm × 7.5 mm) column, operated at 40 °C using a mobile phase of THF at a 1 mL min^–1^ flow rate. The detector used in this equipment was infrared type and the injection volume of the sample was 100 μL. The calibration was performed using polystyrene standards.

##### 2.3.4.2 Evaluation of Characteristics of Lignin-Based Alkyd Resins

Three test methods were chosen to determine if the modification with the kraft lignin fraction was suitable or if any other modification was necessary in the alkyd formulation to achieve the standard characteristics of an anticorrosion coating on metal surfaces. The basic characteristics of the dried films (surface coatings) generated using the lignin-based alkyd resin and an alkyd resin prepared with phthalic anhydride in the original composition were compared in terms of the dry time test, hardness, and gloss. These analytical tests for determination of the characteristics of the surface film were performed following ASTM D1640 (dry time test), ASTM 3928 (gloss test), and ASTM 4366–16 (Persoz hardness test), which are industrial standard procedures for the evaluation of surface coatings. In the dry time test, the samples were prepared by adding 2% of a blend of driers prior to the test.

##### 2.3.4.3 Evaluation of Characteristics of Anticorrosion Coatings Prepared Using Lignin-Based Alkyd Resins

The viscosity was measured with a Cup Ford-4 viscosity cup at 20 °C, and the pH was determined using a pH meter HI-221 (HANNA). To calculate the solid content of a sample of the paint, the sample was introduced in an oven at 150 °C for 2 h (UNE EN ISO 3251). The volatile organic compound content (% VOC) was determined by the RD 117/2003 method. The gloss of a coated surface was measured using a Micro Tri Gloss 4435 (RHOPOINT) glossmeter at 60° geometry (UNE EN ISO 2813), while the thickness of the coating was analyzed with a Megacheck FN (UNE EN ISO 2808) device. The Persoz hardness of the coated surface was measured by the damping duration of a pendulum that swings on the painted surface (UNE EN ISO 1522).

The adhesion of the paint was tested by the UNE EN ISO 2409 method in which 100% adherence (Gt0) was desired. The method was used to also perform a cross-cut test to check if any paint peeling occurred, which is undesired. The impacts, direct and reverse, were made at 60 cm with an impact mass of 20 mm diameter (UNE EN ISO 6272). Blending was carried out with a mandrel of 5 mm (UNE EN ISO 1519) and a cupping at 7 mm (UNE EN ISO 1519). Finally, corrosion resistance was analyzed by a salt spray test (UNE EN ISO 9227). The painted panels were introduced into a chamber and were sprayed continuously with an atomized saline solution at a constant temperature of 35 °C.

## 3 Results and Discussion

A summary of the full compositional analysis of unfractionated kraft lignin is provided in Table 2 below. Unfractionated, dry kraft lignin had a purity of 94.3 wt % with additional components mainly made up of extractives (2.4 wt %), carbohydrates (1.6 wt %), protein (0.1 wt %), and ash (0.9 wt %). The results from elemental analysis (C, H, N, S, O) for protein content, acid hydrolysis, and UV analysis to determine the content of Klason and acid-soluble lignin are given in the Supporting Information (Tables S2–S3).

**Table 2:** Chemical Composition of the Unfractionated Kraft Lignin Material Used in Solvent Fractionation

component	kraft lignin
dry matter, dm (wt %)	94.2
composition of dry matter (wt % of dm)	
insoluble Klason lignin	92.0
acid-soluble lignin	2.4
total lignin (insoluble + soluble)	94.3
extractives	2.4
carbohydrates	1.6
protein	0.1
ash 550 °C	0.9
total	99.4

### 3.1 Solvent Fractionation of Kraft Lignin at the Laboratory Scale

#### 3.1.1 Production of Soluble and Insoluble Lignin by Ethanol/Water Fractionation

**Figure 4:**
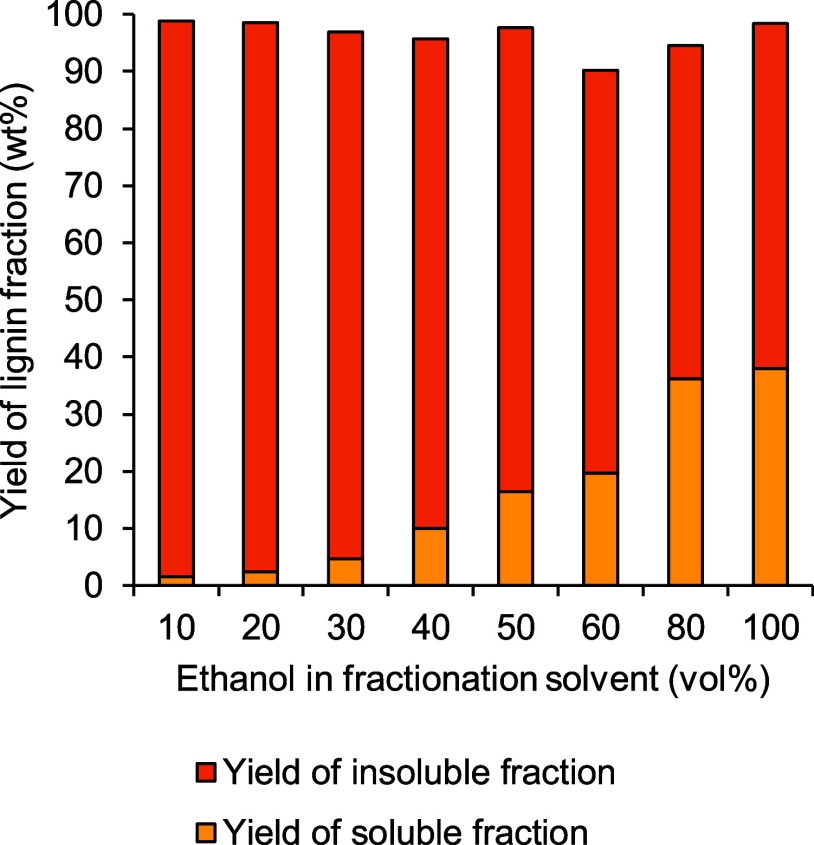
Yields of soluble and insoluble fractions of softwood kraft lignin produced by ethanol/water fractionation as a function of ethanol vol % used in laboratory-scale fractionation.

Yields of soluble and insoluble fractions of kraft lignin produced by fractionation using different ethanol:water ratios are presented in Figure 4.

Overall, the yields of the soluble fraction of lignin increased, while the yields of the insoluble fraction of lignin decreased with an increasing vol % of ethanol in the aqueous solvent. Total combined yields of soluble and insoluble fractions of lignin were nearly 95 wt % or higher for all ratios of ethanol/water used in fractionation except for 60 vol % ethanol. The highest total yield of both fractions of lignin obtained from fractionation with different ratios of ethanol/water was 99 wt %, indicating that 100 wt % mass closure was not achieved for any fractionation condition.

Literature indicates that the yield of soluble lignin can be in a range of 46.8–50 wt % if pure ethanol is used as the solvent, whereas this yield can reach up to 68 wt % if the solvent contained 80:20 vol % ethanol/water in fractionation.^22,26,27^ The yield of the soluble lignin fraction in this study was 36.2 to 38 wt % at 80 to 100 vol % ethanol, which is lower than the expected yields, according to the above literature reports. Nevertheless, the difference could be attributed to a lower purity (e.g., a higher water content) of ethanol, higher lignin/solvent loading, a shorter mixing time, or other unknown differences in the procedure used in this work as compared to the methods in literature.

#### 3.1.2 Molecular Weights and Dispersity of Soluble and Insoluble Lignin Fractions

The average molecular weights, *M*_w_ and *M_n_*, and dispersity of soluble and insoluble fractions of kraft lignin produced by fractionation at different ethanol/water ratios are presented in Table 3. Kraft lignin before fractionation had a *M*_w_ of 4230 g mol^–1^, a *M_n_* of 1000 g mol^–1^, and a dispersity of 4.2, which matches well with the values reported in literature for the molar masses and dispersity of softwood kraft lignin.^26^Table 3 shows that all fractionation conditions using aqueous ethanol as the solvent generated a soluble lignin fraction with the *M*_w_ in the range of 830–2200 g mol^–1^, *M_n_* in the range of 250–500 g mol^–1^, and dispersity in the range of 3.1–4.2 and an insoluble lignin fraction with Mw in the range of 3950–8090 g mol^–1^, *M_n_* in the range of 650–1290 g mol^–1^, and dispersity in the range of 5.3–8.0, indicating an effective fractionation of the original kraft lignin material into two fractions featuring a lower and a higher range of molecular size and dispersity.

**Table 3:** Average Molecular Weights and Dispersity of Unfractionated and Fractionated Kraft Lignin Produced by Solvent Fractionation at the Laboratory Scale

lignin fraction	*M*_w_ (g mol^–1^)		*M_n_* (g mol^–1^)		dispersity	
*unfractionated kraft lignin*^a^	4230		1000		4.2	
	soluble fraction			insoluble fraction		
lignin from EtOH/water fractionation	*M*_w_ (g mol^–1^)	*M_n_* (g mol^–1^)	dispersity	*M*_w_ (g mol^–1^)	*M_n_* (g mol^–1^)	dispersity
EtOH/water (100:0%)	2200	500	4.2	8090	1290	6.3
EtOH/water (80:20%)	1890	470	4.0	6500	850	7.7
EtOH/water (60:40%)	1740	440	4.0	6300	790	8.0
EtOH/water (50:50%)	1750	450	3.9	5500	810	6.8
EtOH/water (40:60%)	1500	440	3.5	4740	890	5.3
EtOH/water (30:70%)	1150	340	3.4	3950	650	6.1
EtOH/water (20:80%)	830	250	3.3	4250	780	5.4
EtOH/water (10:90%)	890	290	3.1	4200	730	5.7

a Unfractionated kraft lignin a was used a raw material containing 94.2 wt % dry matter.

**Figure 5:**
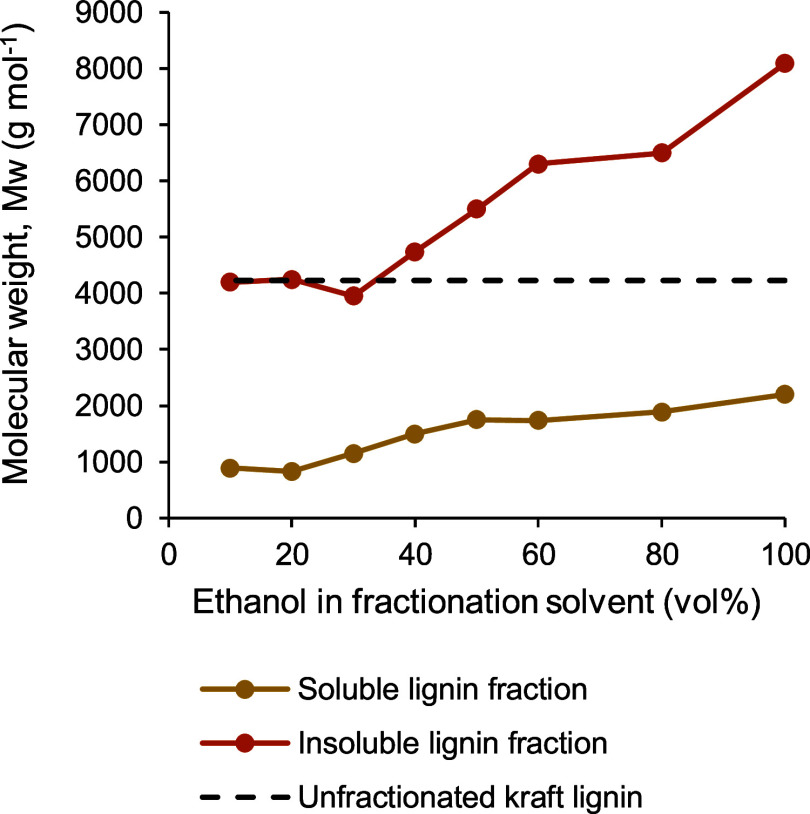
Average molecular weights of soluble and insoluble fractions of softwood kraft lignin produced by ethanol/water fractionation as a function of ethanol vol % used in laboratory-scale fractionation.

Figure 5 illustrates the relationship of the molecular weights (*M*_w_) of soluble and insoluble lignin fractions produced by ethanol/water fractionation as a function of vol % of ethanol in the aqueous solvent. As shown in Figure 5, kraft lignin could be split into two distinct fractions of average molecular weights, one having a *M*_w_ of ≤2200 g mol^–1^, while the other having a *M*_w_ of ≥3950 g mol^–1^, by fractionation in aqueous ethanol. When a higher ethanol vol % was present in the fractionation solvent, the *M*_w_ of the soluble fraction of lignin was generally higher. In fact, the above effect of the solvent ratio on the *M*_w_ of the soluble lignin fraction was highly prominent when 10 to 50 vol % ethanol was employed. The *M*_w_ of the soluble fraction increased more than twice when the content of ethanol in the solvent was raised from 10 to 50 vol % during fractionation. However, the increase in the *M*_w_ of the soluble fraction of lignin was very little (about 9% only) between 50 and 80 vol % ethanol in the fractionation solvent. Clearly, a relatively stable range of the *M*_w_ of the soluble lignin fraction could be produced by using 50 to 80 vol % ethanol during fractionation. An overall similar trend was observed in the case of the *M*_w_ of the insoluble lignin fraction with an increasing ethanol vol % in the fractionation solvent exhibiting a large enhancement of the *M*_w_ below 60 vol % ethanol and a nearly stable *M*_w_ between 60 and 80 vol % ethanol.

Importantly, when pure ethanol was used as the fractionation solvent, the *M*_w_ of both soluble and insoluble fractions of lignin jumped up to 2200 and 8090 g mol^–1^, respectively, which indicates that a fully nonaqueous ethanol medium has strong affinity to the high molar mass of lignin.

Typically, past researchers have demonstrated that aqueous ethanol (80 to 100 vol % ethanol) fractionation of softwood kraft lignin could be effectively divided into two components, one having a lower molecular weight in the range of 1400–2100 g mol^–1^, while the other with a higher molecular weight in the range of 7600–12,117 g mol^–1^, which is well aligned with the results of this present study.^22,26,27^

Duval et al.^22^ has previously reported that the fraction of kraft lignin with a lower molecular weight would be more soluble in a less polar solvent with a lower capability to engage in hydrogen bonding. Since a higher ratio of ethanol to water in their mixture indicates a decrease in the overall polarity and H-bonding capability of the solvent, it can favor the extraction of a fraction of kraft lignin having a lower molecular weight. Thus, this kind of a solvent effect can support the pattern of yields and molecular weights of soluble and insoluble fractions of lignin observed as a function of the ethanol/water ratio in this work.

#### 3.1.3 Chemical Functionalities of Soluble and Insoluble Lignin Fractions

**Figure 6:**
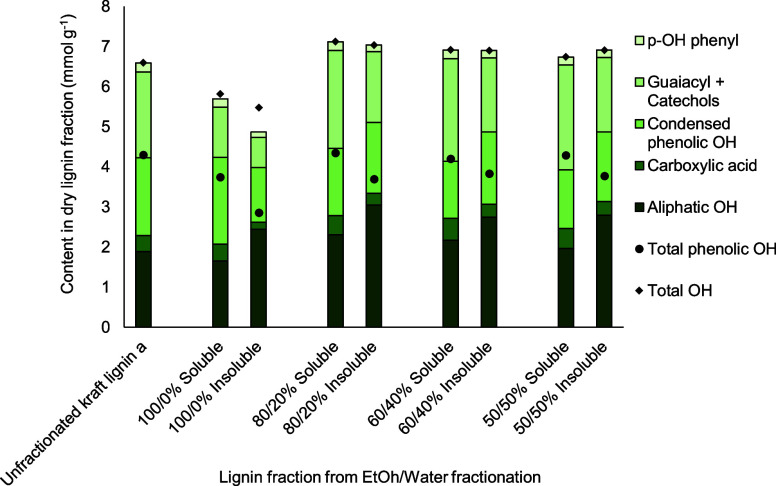
Amounts of different hydroxyl group species (mmol g^–1^) in dry lignin fractionated by different EtOH/water vol % in the laboratory scale. Unfractionated kraft lignin a was used a raw material containing 94.2 wt % dry matter.

Figure 6 demonstrates the distribution of chemical functionalities, mainly different hydroxyl groups, in lignin analyzed for both soluble and insoluble fractions from different ethanol/water fractionation conditions. The soluble fraction contained a lignin rich in total phenolic OH groups and COOH groups, which can be beneficial for alkyd resin preparation. Fractionation with 50 to 80 vol % ethanol could produce soluble lignin fractions with a total phenolic OH group content of 4.20–4.35 mmol g^–1^ and a COOH group content of 0.47–0.55 mmol g^–1^, which is comparable to unfractionated kraft lignin. On the other hand, insoluble fractions contained slightly lower amounts of total phenolic OH groups and COOH groups. Notably, using pure ethanol as the solvent resulted in the biggest difference in the contents of these functional groups between soluble and insoluble lignin fractions. Interestingly, the total OH content was lower for both lignin fractions in the case of pure ethanol, unlike other solvent ratios tested, which could not be fully explained in this study but a larger measurement error in the 100 vol % ethanol case could be a possible factor (Supporting Information, Table S5). Additionally, insoluble lignin was significantly richer in the content of aliphatic OH as compared to the soluble lignin fraction and the unfractionated one.

The compositional differences in chemical functionalities between soluble and insoluble lignin with respect to the raw kraft lignin feedstock were in alignment with past reports in aqueous ethanol or acetone fractionation of softwood kraft lignin.^26,27,36,51^ As conceived by these researchers, the lignin molecules present in the soluble fraction could have undergone more severe degradation during pulping in comparison to the insoluble component, giving rise to a lower average molecular weight, fewer aliphatic hydroxyls, and higher phenolic hydroxyls in these fractions. The concentration of phenolic OH groups is higher in the soluble lignin fraction of a decreasing molecular weight because these materials are degraded more easily due to the cleavage of aryl ether bonds, whereas the aliphatic OH groups are higher in the insoluble lignin fractions of a high molecular weight pointing to less alternation on the lignin side chain or enrichment with carbohydrates.^27^

Figure 6 further reveals the changes occurring in the distribution of the hydroxyl functionalities of kraft lignin after fractionation. The soluble lignin fractions contained a slightly lower (in the case of 50–60 vol % ethanol) or nearly same (80 vol % ethanol) content of condensed phenolic OH, as compared to insoluble lignin after fractionation. This trend was reversed in the case of fractionation with 100 vol % ethanol. On the other hand, the content of the guaiacyl and catechol groups slightly increased in soluble lignin fractions as compared to the insoluble ones for all fractionation conditions. A trend of a lower concentration of aliphatic OH and condensed phenolic OH accompanied by a higher concentration of free phenolic guaiacyl units in soluble lignin has been observed also by other researchers in solvent fractionation.^26,36,51^

Furthermore, as demonstrated by Jääskäläinen et al.,^26^ soluble lignin fractions produced by aqueous ethanol fractionation could contain a greater share of acid-soluble lignin and carbohydrates linked to the lignin structure in comparison to insoluble lignin fractions. This compositional feature could further support the previously observed lower molecular weight and higher concentration of total OH groups in soluble lignin fractions in this study.

In sum, we concluded that the above overall differences in molecular weights, total content, and distribution of OH and COOH groups in soluble and insoluble lignin fractions could contribute together to the observed variation in their solubility in different aqueous ethanol solvent mixtures, therefore leading to a variation in their extraction yields from kraft lignin. As an extension of these observations, we selected the fractionation conditions that employ 50 to 80 vol % ethanol for transferring the process to the pilot scale.

### 3.2 Solvent Fractionation of Kraft Lignin at the Pilot Scale

#### 3.2.1 Production of Soluble and Insoluble Lignin by Ethanol/Water Fractionation

**Figure 7:**
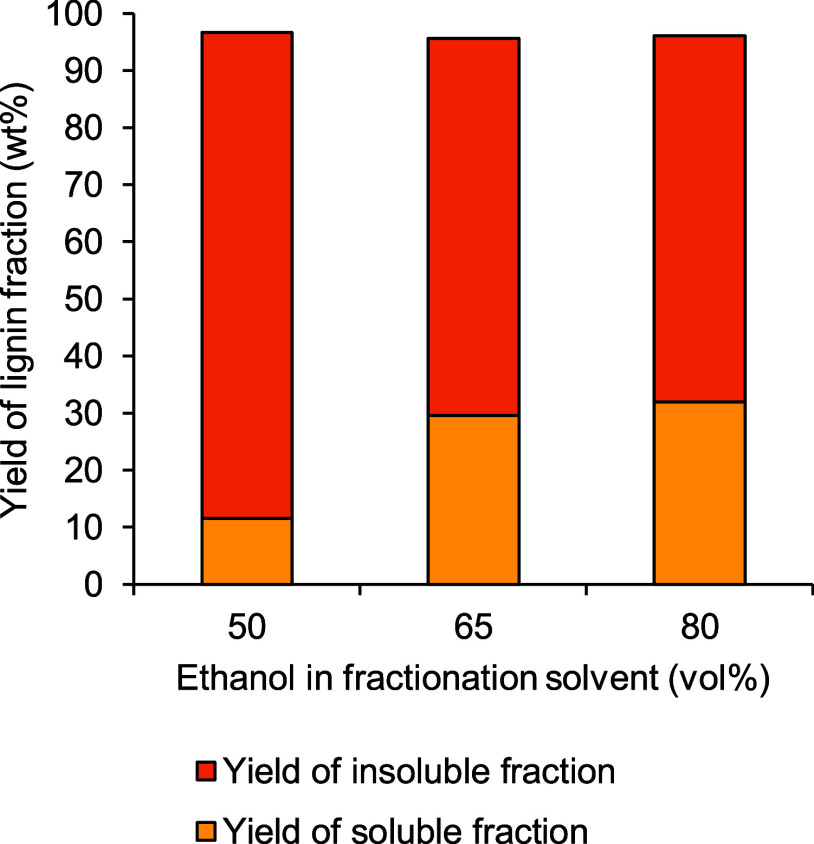
Yields of soluble and insoluble fractions of softwood kraft lignin produced by ethanol/water fractionation as a function of ethanol vol % used in pilot-scale fractionation.

Yields of soluble and insoluble fractions of kraft lignin produced by fractionation using different compositions of the aqueous ethanol solvent are presented in Figure 7. The yield of the soluble lignin fraction could be enhanced significantly from 11.6 to 29.5 wt % by increasing the ethanol content from 50 to 65 vol % in the fractionation solvent. However, the yield of the soluble fraction of lignin increased only up to 31.9 wt % when using 80 vol % ethanol in the solvent. The total yield of soluble and insoluble lignin fractions accounted for 96.1–96.6 wt % of unfractionated kraft lignin. This signifies that the overall trend of yields of soluble and insoluble lignin fractions transferred well from the laboratory to pilot scales. However, the yields of the soluble lignin fraction at the pilot-scale operation suffered a loss of 29 and 12% when fractionating with 50 vol % and 80 vol % ethanol, respectively, in comparison to the laboratory scale. Interestingly, the yield of the insoluble lignin fraction increased by 5 and 10% for 50 and 80 vol % ethanol, respectively, as compared to the laboratory scale. This suggests that the recovery of lignin fractions at the pilot scale was not as efficient as laboratory-scale methods. First, the recovery of the soluble lignin fraction was challenging due to very slow filtration, which might have been attributed to the precipitation of part of soluble lignin in presence of water in the solvent. Additionally, drying the large mass of insoluble solids uniformly and effectively after the washing step was a challenge at the pilot scale. Handling losses also contributed to a lower recovery of soluble lignin fractions. These challenges are important areas where improvement is needed for process intensification of this technology and will be probed in more detail in a future study by the authors.

#### 3.2.2 Molecular Weights and Dispersity of Soluble and Insoluble Fractions

The average molecular weights, *M*_w_ and *M_n_*, and dispersity of soluble and insoluble fractions of kraft lignin produced by different conditions of aqueous ethanol fractionation are listed in Table 4. This ethanol/water fractionation at the pilot scale gave rise to soluble lignin fractions with *M*_w_ in the range of 1930–3000 g mol^–1^, *M_n_* in the range of 395–546 g mol^–1^, and dispersity in the range of 4.1–5.6, and insoluble lignin fractions with *M*_w_ in the range of 6300–8700 g mol^–1^, *M_n_* in the range of 790–1050 g mol^–1^, and dispersity in the range of 7.4–8.3, exhibiting an effective fractionation of the original kraft lignin material into two fractions of lower and higher ranges of molecular weights and dispersity. The above data point to a moderate level of similarity between laboratory- and pilot-scale fractionation in terms of molecular weights and dispersity for the same solvent compositions. Importantly, it was noted that the molecular weights of soluble and insoluble lignin materials were significantly higher for most of the pilot-scale fractionation tests.

**Table 4:** Average Molecular Weights and Dispersity of Unfractionated and Fractionated Kraft Lignin Produced by Solvent Fractionation at the Pilot Scale

lignin fraction	*M*_w_ (g mol^–1^)		*M_n_* (g mol^–1^)		dispersity	
*unfractionated kraft lignin*^a^	4230		1000		4.2	
*unfractionated kraft lignin*^b^	4270		470		9.2	
	soluble fraction			insoluble fraction		
lignin from EtOH/water fractionation	*M*_w_ (g mol^–1^)	*M_n_* (g mol^–1^)	dispersity	*M*_w_ (g mol^–1^)	*M_n_* (g mol^–1^)	dispersity
EtOH/water (80:20%)^a^	3000	546	4.1	8700	1050	8.3
EtOH/water (65:35%)^b^	2239	395	5.6	6300	790	8.0
EtOH/water (50:50%)^a^	1930	470	4.1	6300	860	7.4

a Unfractionated kraft lignin a was used as the raw material containing 94.2 wt % dry matter.

b Unfractionated kraft lignin b was used as the raw material containing 65.0 wt % dry matter.

**Figure 8:**
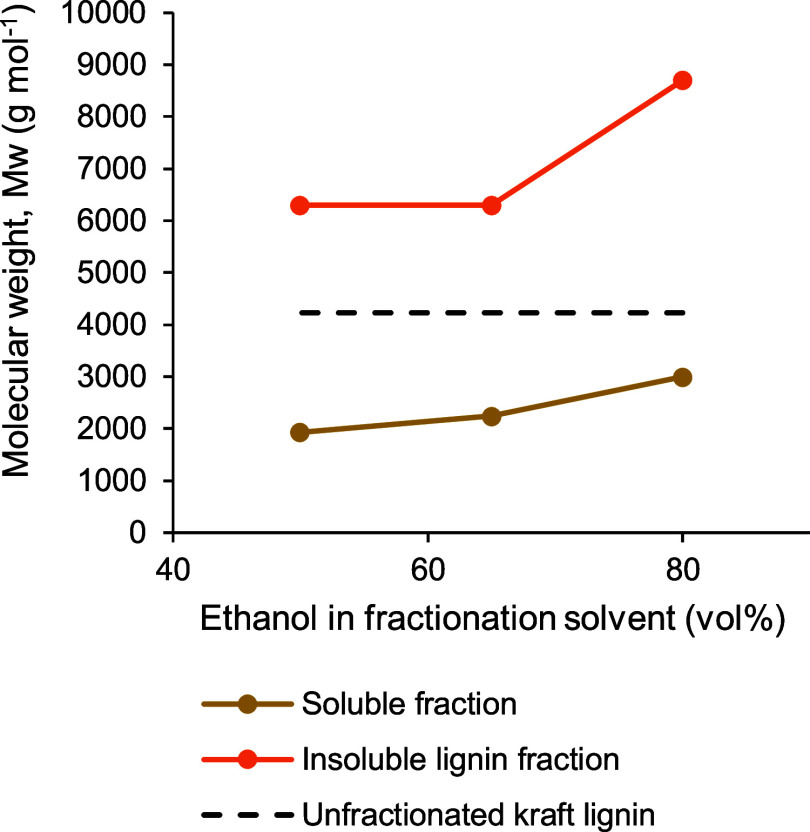
Average molecular weights of soluble and insoluble fractions of softwood kraft lignin produced by ethanol/water fractionation as a function of ethanol vol % used in pilot-scale fractionation.

Figure 8 depicts the relationship of molecular weights of soluble and insoluble fractions with the ethanol content of the fractionation solvent used in the pilot scale. According to Figure 8, the *M*_w_ of both soluble and insoluble fractions of lignin produced in the pilot scale increased with a higher vol % of ethanol in the fractionation solvent. The above data suggest that the overall trend of *M*_w_ and *M_n_* of lignin fractions as a function of ethanol/water ratios of fractionation transferred well from laboratory to pilot scales, while that of dispersity of the lignin fraction was found to be deviating.

#### 3.2.3 Chemical Functionalities of Soluble and Insoluble Lignin Fractions

**Figure 9:**
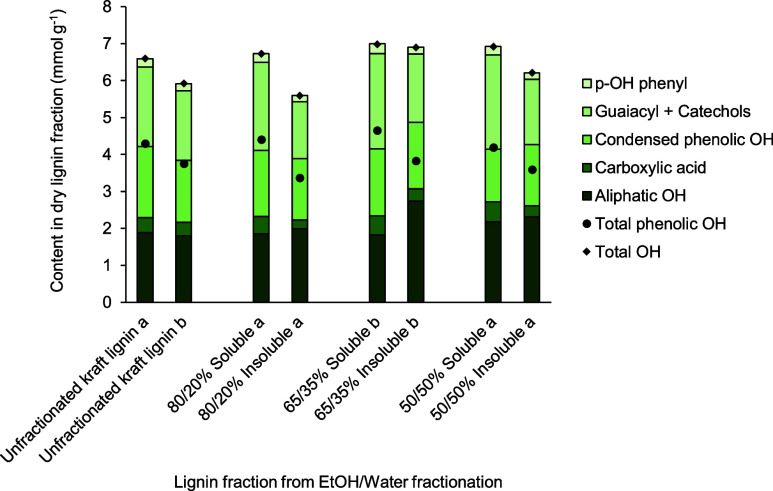
Amounts of different hydroxyl group species (mmol g^–1^) in lignin fractionated by different EtOH/water vol % in the pilot scale. The raw lignin material, unfractionated kraft lignin a containing 94% dry matter, was used for 80 and 50 vol % EtOH fractionation, while the other raw lignin material, unfractionated kraft lignin b containing 65% dry matter, was used for 65 vol % EtOH fractionation.

Figure 9 illustrates the distribution of different chemical functionalities, mainly hydroxyl groups, in soluble and insoluble lignin fractions produced at the pilot scale with 50 to 80 vol % ethanol as the solvent. The content of the total phenolic OH group, total OH groups, and the COOH group ranged from 4.20 to 4.66, 6.73 to 6.99, and 0.47 to 0.55 mmol g^–1^ in the soluble lignin fractions produced at the pilot scale. This indicates that the soluble fractions of lignin at the pilot scale had nearly the same content of total phenolic OH and OH groups but a slightly higher content of COOH groups as compared to unfractionated kraft lignin. Furthermore, insoluble lignin fractions showed a lower content of total phenolic OH and OH groups as well as COOH groups than soluble fractions of lignin for each condition of aqueous ethanol fractionation. In addition, the distribution of these main phenolic OH and COOH groups in soluble lignin fractions did not change significantly with the ratio of ethanol to water in the solvent at the pilot scale. Hence, it is evident that the overall chemical functionalities of soluble and insoluble lignin products of ethanol/water fractionation at the pilot scale were in alignment with those observed at the laboratory scale.

In addition, some differences in the ash content of soluble lignin were also observed, depending on the ratio of ethanol and water used in the fractionation solvent. Soluble lignin produced in 80 and 65 vol % EtOH contained 1.1 and 0.9 wt % ash, respectively, whereas 50 vol % EtOH-soluble lignin had 2.40 wt % ash. Typically, a lower mineral content in the lignin intermediate is desired as it helps in preventing phase separation in alkyd resin production as per best practices in the paint and coating industry.

In sum, it was concluded that the fractionation of kraft lignin in the laboratory could be effectively scaled up to the pilot scale without a significant loss of yields, molecular weights, and chemical functionalities. Due to a lower molar mass (≤3000 g mol^–1^), lower dispersity (≤5.6), and a higher content of phenolic OH and COOH groups, the soluble lignin fractions from the pilot-scale process were chosen as the lignin-based polyacid intermediates to be utilized in the preparation of the alkyd resin formulation.

### 3.3 Coating Applications of Lignin-Based Alkyd Resins

#### 3.3.1 Characteristics of the Lignin-Based Alkyd Resin Formulation

The alkyd resin formulation could be successfully prepared using 65 and 80 vol % EtOH-soluble lignin fractions of kraft lignin obtained by pilot-scale fractionation. These two soluble lignin materials produced alkyd coatings with similar characteristics. Since the yield of 80 vol % EtOH-soluble lignin is the highest among all soluble lignin products of pilot-scale fractionation, we concluded that the full analysis of the alkyd resin and coatings prepared using this soluble lignin will be the most relevant case study for industrialization of the lignin-based alkyd coating process. It is important to note that 50 vol % EtOH-soluble lignin produced at the pilot scale failed to generate an alkyd resin suitable for coating preparation because this lignin material precipitated in the esterification reactor. The reason for this precipitation is not clear, but we hypothesize that the ash impurity (and Na content) in this lignin fraction could have contributed to polymerization of the material in the esterification step. Further in-depth investigation is necessary to evaluate the above reaction pathway of 50 vol % EtOH-soluble lignin, which was out of the scope of this study.

The alkyd resin prepared using the 80% EtOH-soluble lignin product of pilot-scale fractionation had a solid content of 41.6 wt %, a pH of 8.7, and viscosity of 1200 cps at 25 °C. Based on the data listed in Table 5 and literature reports, it was concluded that the difference between the molecular weight and dispersity of the lignin-based alkyd resin and the standard alkyd resin was within an acceptable range,^14^ which indicated that the lignin-based alkyd resin was suitable for testing in the paint formulation.

**Table 5:** Characteristics of Surface Coatings Prepared from the Alkyd Resin Formulation

resin properties	standard alkyd resin	lignin-based alkyd resin
Molecular Weight and Dispersity of Resin		
*M*_w_ (g mol^–1^)	4650	4110
*M*_n_ (g mol^–1^)	1660	1430
dispersity	2.80	2.90
Dry Time Test Characteristics		
set-to-touch time (min)	20	22
dust-free time (min)	25	31
tack-free time (min)	126	143
dry through time (hours)	72	87
Gloss Test Characteristics (ASTM 3928)		
gloss 60° (gloss)	89	90
Persoz Hardness Test Characteristics (ASTM 4366–16)		
Persoz hardness after 1 week of drying (s)	63	55

Furthermore, according to Table 5, the lignin-based alkyd resin showed adequately similar properties during the dry time test, gloss tests, and Persoz hardness tests as compared to the standard alkyd resin, which suggests that the modification of the alkyd resin with the lignin fraction had no significant effect on the main properties of the film.

#### 3.3.2 Characteristics of Anticorrosion Metal Coatings Prepared Using Lignin-Based Alkyd Resins

The effect of different proportions of the melamine resin and the lignin-based bioalkyd resin was studied under different cured conditions to achieve a good balance of coating properties, in comparison to the commercial oil-based paints. Table 6 summarizes the characteristics of the Persoz hardness and gloss of these coatings prepared using lignin-based alkyd resins. A Persoz hardness greater than 180 s and gloss more than 60% were set as desired properties for the biocoatings.

**Table 6:** Persoz Hardness and Gloss Characteristics of the Lignin-Based Alkyd Coatings Prepared at Different Ratios of the Melamine Resin and the Bioalkyd Resin and Different Curing Conditions

	Persoz hardness (s), gloss (GU)					
	140 °C		150 °C		160 °C	
resin ratio (bioalkyd-to-melamine)	30 min	60 min	30 min	60 min	30 min	60 min
8.8	99	143	136	158	137	160
7.5	87	136	132	169	157	180
6.9	106, 82	151, 76	140, 77	180, 72	166, 73	188, 67

Table 6 indicates that the lignin-based biocoating produced using the ratio of the bioalkyd/melamine resin as 6.9 showed the best results of Persoz hardness. An increase in the curing temperature up to 150–160 °C and a curing time of 60 min could help achieve a Persoz hardness of 180 s or more. However, an increase of the curing temperature and curing time decreased the gloss. Although at 160 °C and 60 min curing, a higher Persoz hardness was attained (188 s), the test at 150 °C and 60 min showed a better balance of Persoz hardness and gloss.

**Figure 10:**
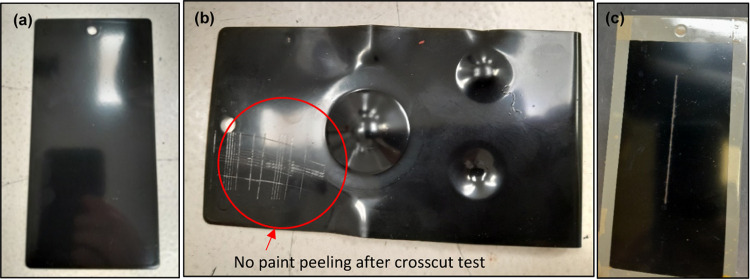
Carbon steel panel (a) after application of the lignin-based biocoating prepared with the bioalkyd-to-melamine resin ratio of 6.9 and curing at 150 °C and 60 min by a spray gun, (b) after physical tests of adhesion, impact, blending, and cupping, and (c) after the salt spray test.

Figure 10 shows metal surfaces that underwent a biocoating application and tests. The surface was cleaned, degreased, and pickled prior to coating application. It was painted using a spray gun with a nozzle of 1.4–2 mm at a pressure of 40–60 bar.

Figure 10 shows the results of applying the lignin-based biocoating on the carbon steel surface and the subsequent physical tests performed on this coated surface. From the above tests, 100% adhesion (Gt0) of the paint was observed and no paint peeling in the cross-cut test occurred in the case of the lignin-based biocoating. No cracks or peeling was found in these tests, which suggests that the biocoating developed with the lignin-based alkyd resin had good physical resistances compared to the commercial standard product. Furthermore, no corrosion or blisters appeared after 120 h of the salt spray test.

As listed in Table 7, the characteristics of the lignin-based alkyd coating prepared with the bioalkyd-to-melamine resin ratio of 6.9, cured at 150 °C for 60 min, and applied by a spray gun were mostly comparable with that of the commercial standard alkyd coating in terms of the pH, solid content, thickness, gloss, hardness, and physical and salt spray tests. The viscosity of the lignin-based coating was significantly higher but this did not cause any problem in coating application. Moreover, the lignin-based alkyd coating showed a reduction in VOC % and an increase in the biocontent, which are highly desired.

**Table 7:** Characteristics of a Commercial Standard Alkyd Coating and Lignin-Based Alkyd Coating Prepared with the Bioalkyd-to-Melamine Resin Ratio of 6.9, Cured at 150°C, 60 min, and Applied by a Spray Gun

characteristics	commercial standard coating	lignin-based biocoating
viscosity (Cup Ford-4 at 20 °C, s)	55 ± 5	>150
pH	8.2–8.5	8.2
solids (wt %)	50 ± 5	47
volatile organic compounds (% VOC)	5	1.66
biocontent (%)	5	10.8
gloss (GU)	>60	72
physical tests	OK	OK
thickness (μm)	30–40	30–40
Persoz hardness (s)	180	180
salt spray test	120 h (OK)	120 h (OK)

Together, all of the above results, as presented in Figure 10 and Table 7, indicated that the biocoating produced from the lignin-based alkyd resin at optimal conditions showed a good balance of physical properties, mechanical strength, and corrosion resistance, thus matching well in overall performance characteristics of the commercial standard coating.

### 3.4 Valorization of the Insoluble Lignin Fraction to Dispersants for Special Carbon Black

**Figure 11:**
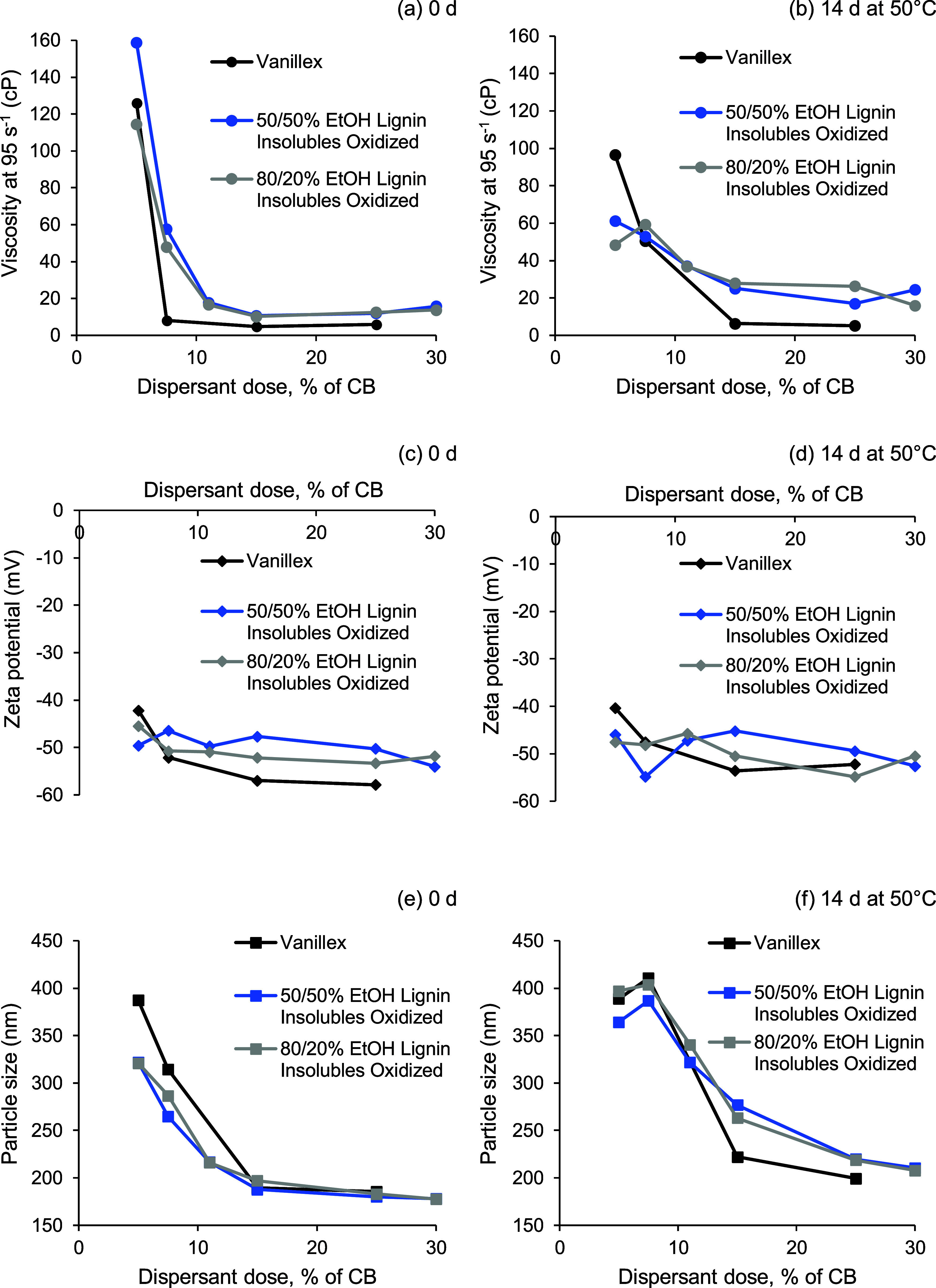
Evaluation of aqueous dispersions of special carbon black with the dispersants. Viscosity measured at a 95 s^–1^ shear rate after storage for (a) 0 days and (b) 14 days at 50 °C; ζ-potential after storage for (c) 0 days and (d) 14 days at 50 °C; and average particle size after storage for (e) 0 days and (f) 14 days at 50 °C as a function of the dispersant dosage.

The insoluble lignin fractions produced by pilot-scale solvent fractionation were further oxidized and evaluated as a dispersant product for special carbon black (CB). The viscosity of the CB paste dispersed using oxidized insoluble lignin from solvent fractionation with 80 and 50 vol % EtOH as a function dispersant dosing is presented in Figure 11a. The dispersing ability of the oxidized insoluble fraction is compared to Vanillex, which is a commercial modified lignosulfonate dispersant. Figure 11b,c demonstrate the corresponding ζ-potential and particle size of these materials, respectively. The oxidized insoluble lignin fraction exhibited a good performance against the commercially available product (Vanillex).

From the above results, it was concluded that the kraft lignin raw material in this work could be valorized fully. While the soluble lignin fraction was found to have favorable properties to be utilized as an alkyd resin formulation for coating production, the insoluble fraction could be more useful in dispersant applications. This study also demonstrated that the insoluble fractions of kraft lignin, not only unfractionated kraft lignin, can be oxidized effectively to produce dispersants.

### 3.5 Prospects and Outlook

In this work, a few aspects of fractionation of kraft lignin by ethanol/water for alkyd resin production were identified that require further investigation. Future research work should focus on these issues to close the gaps in the current knowledge generated from this study. First, the effect of lignin loading in ethanol/water and ethanol recovery/recycling should be evaluated as this is an important factor to understand the economics of the process at scale. Second, the challenges of filtration and drying at the large scale should be further investigated to increase the economic feasibility of lignin fractionation. Besides, the flexibility and weather resistance of lignin-based alkyd coatings, volatile organics release for the odor profile, and pH-dependent solubility of the soluble lignin fraction should be explored to gain more insights into their practical applications, which was outside the scope of this study. New research work is also encouraged to explore the underlying reaction mechanism of the production of the alkyd resin from fractionated lignin materials. It would be beneficial to investigate if the soluble lignin fraction can act as both polyacid and polyol substitutes in the alkyd resin formulation. The findings from this type of research could hold key insights about how the process design as well as the performance of the resins/coatings can be improved and suggest pathways to enhance the lignin content in the alkyd resin. Finally, technoeconomic analysis and life-cycle analysis of the lignin-based alkyd resin should be performed to check the economic feasibility of bringing these new sustainable products to the biobased industry market, specifically paint and coating sectors, and also to assess their impact on the environment.

## Conclusions

This study presents a new approach for valorization of softwood kraft lignin into a uniform, low-molecular-weight, soluble fraction to be used as a substitute of petroleum-derived phthalic anhydride in the formulation of alkyd resins to produce surface coatings. In a simple, mild, one-step fractionation method using aqueous ethanol, two distinguishable lignin fractions, one with a lower molecular weight of ≤2200 g mol^–1^ and the other with a higher molecular weight of approximately ≥3950 g mol^–1^, were produced. The soluble fraction of lignin showed increasing yields and molecular weights as a function of the ethanol/water ratios employed in fractionation. The insoluble fraction, on the other hand, showed the opposite trend of yields and molecular weights. The soluble fraction, characterized as a low-molecular-weight, homogeneous material with a relatively high concentration of phenolic hydroxyl and carboxylic acid groups, was therefore subsequently chosen for testing as a biobased polyacid/polyol substrate in the preparation of alkyd resins. The insoluble fraction could also be further valorized by alkali-O_2_ oxidation to produce a dispersant for special carbon black pigments. In this study, a relatively greener, biobased, and low-cost solvent, ethanol, has been optimized for efficient fractionation of kraft lignin, making downstream separation and drying stages relatively easy and cost-effective. Furthermore, this process has been successfully tested at the pilot scale, for the first time according to our knowledge, to fully valorize kraft lignin to generate lignin-based intermediates—a primary soluble lignin product for bioalkyd resin, and a dispersant byproduct for special carbon black. The bioalkyd resin was further successfully utilized to produce a biobased anticorrosion coating on a metal surface, exhibiting good characteristics and performance against a commercial standard.

## Supporting Information Available

Additional experimental data including laboratory analytical results, spectra, and chromatographs (PDF)
