# Supplementary material for: Fractionation of Kraft Lignin for Production of Alkyd Resins for Biobased Coatings with Oxidized Lignin Dispersants as a Co-Product
Source: ACS Omega. 2024 Nov 4;9(46):46276–92. doi: 10.1021/acsomega.4c07187 (PMC11579782; doi:10.1021/acsomega.4c07187)
Supplement: Supplementary file 1 — ao4c07187_si_001.pdf [file ao4c07187_si_001.pdf]

Supporting Information: Additional Experimental Data

Fractionation of Kraft Lignin for Production of Alkyd Resin for Bio-based Coatings with Oxidized Lignin Dispersants as a Co-Product

Arpa Ghosh[1]*, Olesya Fearon[1,2], Melissa Agustin[1], Susana Alonso[3], Estefanía Cámara Balda[3], Saulo Franco[3], Anna Kalliola[1]

[1]VTT Technical Research Centre of Finland Ltd., P.O. Box 1000, FI-02044 VTT Espoo, Finland

[2]Present address: Metsä Fibre, Revontulenpuisto 2, 02100 Espoo, Finland

[3]Barpimo S.A., Calle San Fernando, 116, 26300 Nájera, La Rioja, España

*Corresponding author. E-mail address of corresponding author: arpa.ghosh@vtt.fi

Table S1. Reproducibility of yields of lignin fractions produced by fractionation with 50/50 vol% EtOH/Water in laboratory scale.

Lignin fraction	Replicate – i Yield (wt%)	Replicate – ii Yield (wt%)	Average of Yield (wt%)	Std. Dev of Yield (wt%)
50 vol% EtOH soluble lignin	15.6	17.1	16.36	1.03
50 vol% EtOH insoluble lignin	81.9	80.5	81.21	0.98

Table S2. Elemental analysis of unfractionated kraft lignin

Element	Composition (%)
Nitrogen	0.02
Carbon	64.7
Hydrogen	5.7
Sulfur	1.7
Oxygen	24.0
Total	96.2

Table S3. Klason and acid-soluble lignin in unfractionated kraft lignin by NREL procedure and UV analysis

Replicate	1	2	
Dry matter content [%]	94.24934	94.22785	
Average dry matter content [%]	94.2386		
Extract content [%]	2.409589		
**DRY MATTER CONTENT AFTER EXTRACTION**			
Dry matter content [%]	95.86751	95.87629	
Average dry matter content [%]	95.8719		
**ACID HYDROLYSIS**			
Replicate	1	2	3
Extracted mass in [g]	0.3001	0.3012	0.3005
**GRAVIMETRIC LIGNIN**			
Replicate	1	2	3
Mass of precipitate [g]	0.2713	0.2725	0.2711
Gravimetric Lignin %	92.0237	92.0932	91.8334
Gravimetric lignin average [%]	91.9834		
**SOLUBLE LIGNIN** (Goldsmith, 1971)			
V (sample) [l]	0.1		
d [cm]	1		
Replicate	1	2	3
Dilution factor for wavelength 215 nm	10	10	10
Absorbance at 215 nm A	0.516	0.513	0.499
Absorbance at 215 nm B	0.522	0.507	0.494
Dilution factor for wavelength 280 nm	10	10	10
Absorbance at 280 nm A	0.218	0.221	0.212
Absorbance at 280 nm B	0.222	0.217	0.21
Soluble lignin [g/l]	0.0710	0.0697	0.0679
Soluble lignin [%]	2.4095	2.3559	2.3014
Soluble lignin average [%]	2.3556		
Total lignin [%]	94.3390		

Table S4. SEC measurements of molecular weights and dispersity of lignin fractions produced by different EtOH/Water vol% in laboratory scale. Measurements are presented as Average ± Std. Dev. in table.

Lignin fraction from EtOH/Water vol% fractionation	Mw* (g/mol)	Mn* (g/mol)	Dispersity
80/20% Soluble	1890±6	474±15	4.0±0.1
60/40% Soluble	1743±13	439±3	4.0±0.0
50/50% Soluble	1745±49	448±17	3.9±0.0

*Raw values of Mw and Mn obtained from the instrument were rounded up to their closest value as a multiple of 10 in the tables/figures in main manuscript (e.g. 1743 g/mol rounded to 1740 g/mol, and 448 g/mol rounded to 450 g/mol etc.).

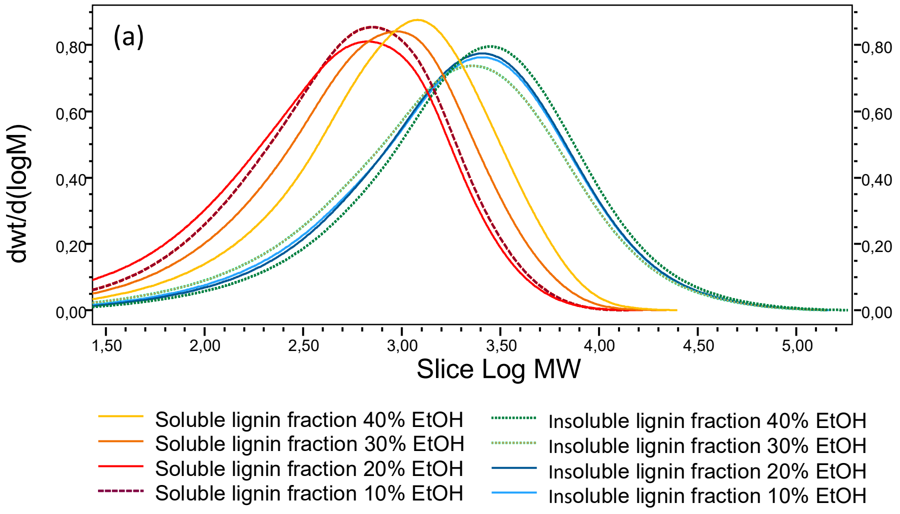

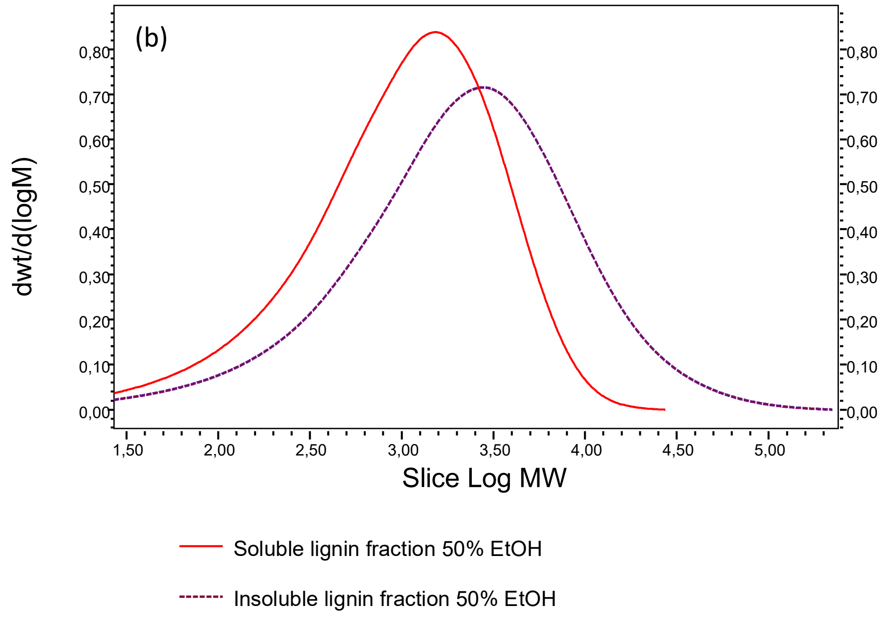

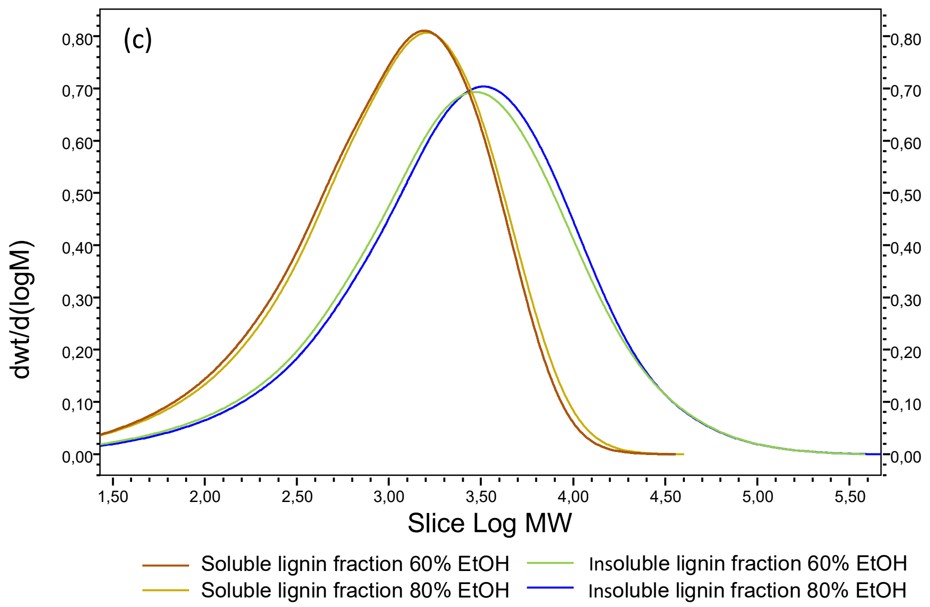

Figure S1. SEC chromatographs of different soluble and insoluble lignin fractions produced by fractionation of kraft lignin using (a) 10 – 40 vol% ethanol/water, (b) 50 vol% ethanol/water, (c) 60 – 80 vol% ethanol/water at laboratory scale.

Table S5. Amounts of different hydroxyl group species (mmol g^{-1}) in dry lignin fractionated by different EtOH/Water vol% in laboratory scale. Measurements are presented as Average ± Std. Dev. in table.

Lignin fraction from EtOH/Water vol% fractionation	Aliphatic OH	COOH	Condensed PhOH	Guaiacyl + Catechols	p-OH phenyl	Total PhOH	Total OH
Unfractionated kraft lignin [a]	1.89±0.03	0.40±0.01	1.93±0.08	2.15±0.06	0.22±0.01	4.30±0.18	6.60±0.22
100/0% Soluble	1.65±0.05	0.42±0.01	2.16±0.18	1.26±0.12	0.20±0.01	3.75±0.36	5.82±0.41
100/0% Insoluble	2.44±0.05	0.18±0.01	1.36±0.01	0.75±0.00	0.14±0.00	2.86±0.06	5.48±0.12
80/20% Soluble	2.31±0.03	0.47±0.02	1.68±0.06	2.44±0.07	0.22±0.00	4.35±0.13	7.13±0.18
80/20% Insoluble	3.05±0.06	0.29±0.01	1.77±0.05	1.76±0.06	0.17±0.02	3.70±0.15	7.04±0.21
60/40% Soluble	2.17±0.02	0.55±0.00	1.42±0.07	2.56±0.09	0.21±0.02	4.20±0.19	6.92±0.21
60/40% Insoluble	2.74±0.01	0.33±0.00	1.80±0.02	1.85±0.06	0.18±0.00	3.83±0.12	6.90±0.13
50/50% Soluble	1.97±0.08	0.49±0.02	1.46±0.08	2.62±0.05	0.20±0.01	4.29±0.20	6.75±0.29
50/50% Insoluble	2.79±0.02	0.34±0.00	1.74±0.02	1.86±0.07	0.18±0.01	3.78±0.14	6.91±0.17

a: Unfractionated kraft lignin a was used as raw material containing 94.2 wt% dry matter.

Table S6. Amounts of different hydroxyl group species (mmol g^{-1}) in dry lignin fractionated by different EtOH/Water vol% in pilot scale. Measurements are presented as Average ± Std. Dev. in table.

Lignin fraction from EtOH/Water vol% fractionation	Aliphatic OH	COOH	Condensed PhOH	Guaiacyl + Catechols	p-OH phenyl	Total PhOH	Total OH
Unfractionated kraft lignin [a]	1.89±0.03	0.40±0.01	1.93±0.08	2.15±0.06	0.22±0.01	4.30±0.18	6.60±0.22
Unfractionated kraft lignin [b]	1.80±0.05	0.36±0.01	1.68±0.04	1.89±0.03	0.19±0.01	3.76±0.08	5.93±0.14
80/20% Soluble [a]	1.85±0.00	0.47±0.01	1.79±0.04	2.38±0.06	0.24±0.00	4.41±0.10	6.73±0.11
80/20% Insoluble [a]	1.99±0.05	0.24±0.01	1.66±0.01	1.54±0.01	0.17±0.01	3.37±0.03	5.60±0.09
65/35% Soluble [b]	1.82±0.01	0.51±0.01	1.82±0.02	2.58±0.07	0.27±0.04	4.66±0.12	6.99±0.14
65/35% Insoluble [b]	2.74±0.01	0.33±0.00	1.80±0.02	1.85±0.06	0.18±0.00	3.83±0.12	6.90±0.13
50/50% Soluble [a]	2.17±0.02	0.55±0.00	1.42±0.07	2.56±0.09	0.22±0.03	4.20±0.19	6.92±0.21
50/50% Insoluble [a]	2.31±0.01	0.30±0.00	1.66±0.03	1.76±0.03	0.18±0.00	3.60±0.05	6.22±0.06

a: Unfractionated kraft lignin a was used as raw material containing 94.2 wt% dry matter.
b: Unfractionated kraft lignin b was used as raw material containing 65.0 wt% dry matter.

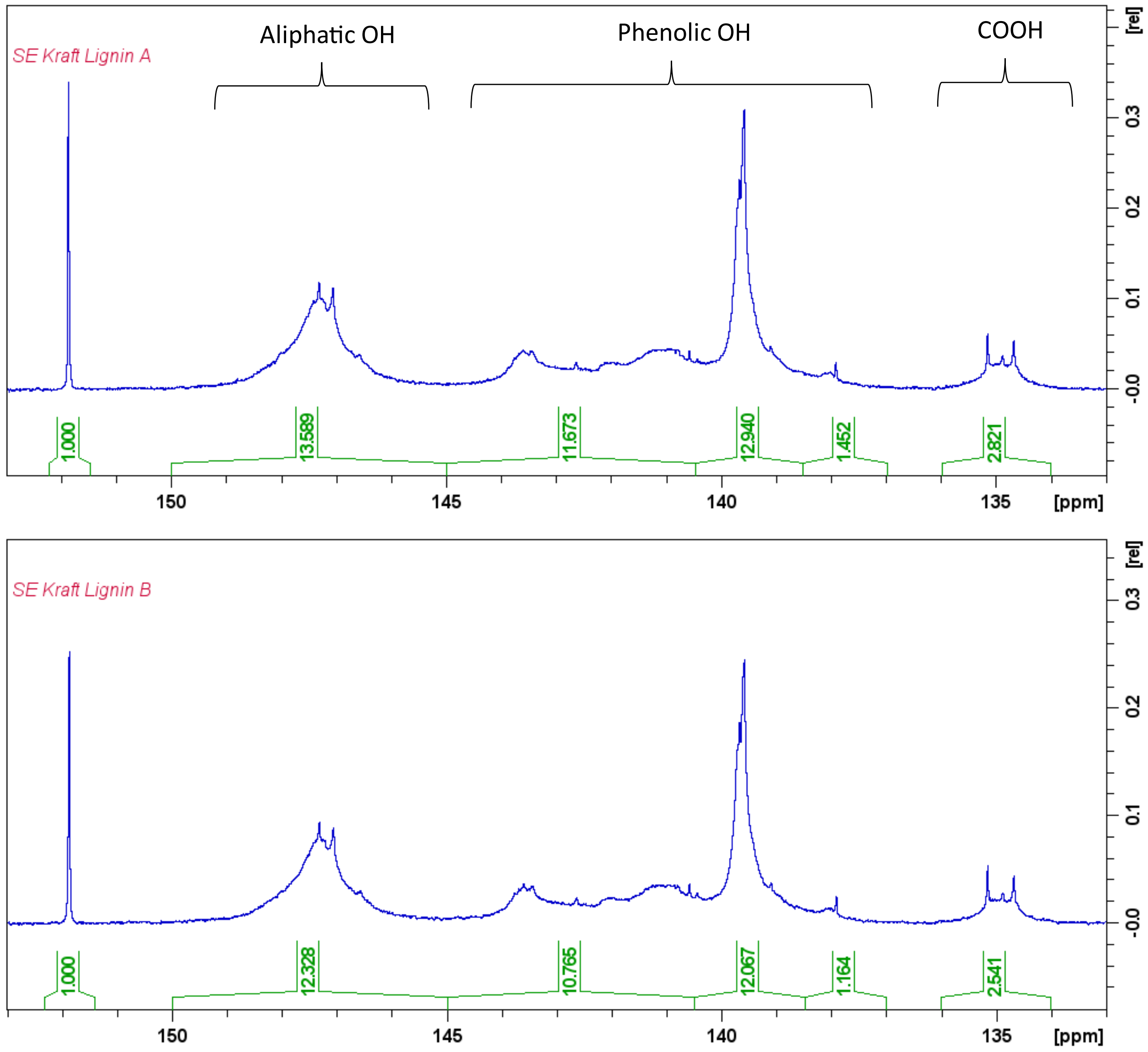

Figure S2. ^{31}P-NMR spectra of unfractionated kraft lignin. SE = Stora Enso, kraft lignin provider for this work. A and B indicate the two replicates of the measurements.
